# Citrullination of NF‐κB p65 by PAD2 as a Novel Therapeutic Target for Modulating Macrophage Polarization in Acute Lung Injury

**DOI:** 10.1002/advs.202413253

**Published:** 2025-03-14

**Authors:** Xin Yu, Yujing Song, Tao Dong, Wenlu Ouyang, Chao Quan, Liujiazi Shao, Leonard Barasa, Paul R. Thompson, Mao Zhang, Jianjie Ma, Katsuo Kurabayashi, Yongqing Li

**Affiliations:** ^1^ Department of Surgery University of Michigan Health System Ann Arbor MI 48109 USA; ^2^ Department of Emergency Medicine Second Affiliated Hospital Zhejiang University School of Medicine No.88 Jiefang Road Hangzhou Zhejiang 310009 China; ^3^ Department of Mechanical and Aerospace Engineering New York University Tandon School of Engineering Brooklyn NY 11201 USA; ^4^ Department of Mechanical Engineering University of Michigan Ann Arbor MI 48109 USA; ^5^ Department of Physiology Xuzhou Medical University Xu Zhou Jiangsu 221002 China; ^6^ Department of Metabolism and Endocrinology The Second Xiangya Hospital Changsha 410011 China; ^7^ Department of Urology The Xiangya Hospital Changsha 410013 China; ^8^ Department of Anesthesiology Beijing Friendship Hospital Capital Medical University No.95 Yong‐an Road, Xicheng District Beijing 100050 China; ^9^ Program in Chemical Biology, Department of Biochemistry and Molecular Biotechnology University of Massachusetts Chan Medical School Worcester MA 01605 USA; ^10^ Department of Surgery Division of Surgical Science University of Virginia Charlottesville VA 22903 USA; ^11^ Department of Chemical and Biomolecular Engineering New York University Tandon School of Engineering Brooklyn NY 11201 USA

**Keywords:** acute lung injury, gold nanoparticles, ICAM‐1, macrophage polarization, NF‐κB p65, PAD2

## Abstract

Mediating protein citrullination, peptidyl arginine deiminase 2 (PAD2) has recently been reported to influence macrophage phenotypes. However, the mechanisms of PAD2 on macrophage function in *Pseudomonas aeruginosa* (PA)‐induced acute lung injury syndrome (ALI) remains unclear. Utilizing single‐cell RNA sequencing and mass spectrometry‐based proteomics, a new citrullination site at arginine 171 (R171) is discovered within nuclear factor‐ κB (NF‐κB) p65 catalyzed by PAD2, which modulates PAD2‐NF‐κB p65‐importin α3 pathway and its downstream M1/M2 macrophage polarization. Building on these findings, a cell‐specific targeted therapeutic strategy using gold nanoparticles (AuNPs) conjugated with a novel PAD2 inhibitor, AFM41a, and an intercellular adhesion molecule‐1 (ICAM‐1) antibody is developed. This approach enables the selective delivery of the inhibitor to M1‐polarized macrophages in the PA‐infected alveolar niche. In vivo, this nanomedicine reduces excessive inflammation and promotes M1‐to‐M2 polarization to inhibit ALI. This study highlights the role of PAD2‐mediated citrullination in macrophage polarization and introduces a promising nanoparticle‐based therapy for PA‐induced ALI.

## Graphical abstract

Peptidyl arginine deiminase 2 (PAD2) plays a key role in regulating macrophage function in *Pseudomonas aeruginosa*‐induced acute lung injury (ALI). Using single‐cell RNA sequencing and proteomics, a new PAD2‐catalyzed citrullination site on NF‐κB p65 (171 Arginine), modulating macrophage polarization is identified. A nanoparticle‐based PAD2 inhibitor therapy is developed, reducing inflammation and improving ALI outcomes.

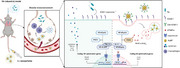

## 1 Introduction

Acute lung injury and acute respiratory distress syndrome (ALI/ARDS) are marked by diffuse alveolar damage and inflammation, posing significant global health and resource challenges.^[^ ^1^, ^2^ ^]^ Pneumonia caused by bacterial and viral infections remains a major risk factor for the development ALI.^[^ ^3^, ^4^ ^]^ *Pseudomonas aeruginosa* (PA), a Gram‐negative pathogen with increasing multidrug resistance (MDR), is frequently associated with hospital‐acquired pneumonia, particularly in immunocompromised patients, leading to ALI and high mortality rates.^[^ ^5^, ^6^ ^]^ Due to the high prevalence of MDR in PA infections and the lack of effective treatments,^[^ ^7^, ^8^ ^]^ it is necessary to develop novel non‐antibiotic therapeutics for PA‐induced ALI.

Peptidylarginine deiminases (PAD, also known as PADI) is an enzyme family involved in the citrullination of arginine residues on proteins, a post‐translational modification that alters protein function and cellular processes.^[^ ^9^ ^]^ Among them, PAD2 is abundantly expressed in myeloid‐derived immune cells, and elevated levels of PAD2 have been reported in the bronchoalveolar lavage fluid (BALF) and serum of ALI patients.^[^ ^10^, ^11^ ^]^ Our recent studies showed that the absence of PAD2 improves survival and alleviates ALI in septic mice.^[^ ^11^, ^12^ ^]^ In vitro experiments revealed that using a pan‐PAD inhibitor, BB‐Cl‐amidine, or silencing the *Pad2* gene can attenuate the polarization of THP‐1 cells toward the M1 phenotype while promoting activation toward the M2 phenotype.^[^ ^13^ ^]^ However, the role of PAD2‐mediated macrophage polarization in PA‐induced ALI has not yet been studied in vivo.

An in vitro study demonstrated that PAD2 can directly citrullinate nuclear factor‐ κB (NF‐κB) p65, leading to reduced expression of NF‐κB‐targeted pro‐inflammatory mediators in *Pad2* deficient macrophages following lipopolysaccharide (LPS) stimulation.^[^ ^14^ ^]^ In contrast, another in vitro study presented conflicting evidence, suggesting that overexpression of PAD2 may inhibit NF‐κB activity by citrullinating the inhibitor of κB (IκB) kinase γ subunit, which facilitates NF‐κB dissociation from IκBα and subsequent nuclear translocation.^[^ ^15^ ^]^ Without in vivo studies, the exact mechanisms by which PAD2 modulates the NF‐κB pathway and influences macrophage phenotypes remain poorly understood, limiting our understanding of these processes in PA‐induced ALI and their potential for advancing therapeutic strategies.

To address these issues, we utilized single‐cell RNA sequencing (scRNA‐seq) to characterize immune cell heterogeneity in the alveolar microenvironment of PA‐induced ALI models and explored PAD2‐dependent cellular and gene changes, specifically focusing on macrophage polarization. Additionally, we employed mass spectrometry‐based proteomics analysis to map specific PAD2‐mediated citrullinated proteins and citrullination modification sites.^[^ ^16^, ^17^ ^]^ Importantly, we identified a specific citrullination site within NF‐κB p65 catalyzed by PAD2, which modulates NF‐κB p65 activity and consequently affects the M1/M2 polarization transition.

Building on these observations, we developed a dual‐function anti‐inflammatory nanomedicine for treating ALI. Previous studies showed that treatment with AFM32a, a small‐molecule PAD2 inhibitor, could alleviate lung injury and improve survival in murine models of LPS‐induced and cecal ligation puncture‐induced sepsis.^[^ ^11^, ^18^ ^]^ However, in vivo administration of small molecules like AFM32a is often limited by rapid metabolism, poor bioavailability, and high off‐target toxicity.^[^ ^19^ ^]^ To address these challenges, we functionalized each AuNP to simultaneously carry a novel PAD2 inhibitor AFM41a^[^ ^20^ ^]^ and intercellular adhesion molecule‐1 (ICAM‐1) antibodies, enabling the AuNPs to cross the pulmonary endothelial barrier and target the PA‐infected alveolar niche.^[^ ^21^, ^22^ ^]^ AuNPs are potent tools for targeted drug delivery due to their biocompatibility, non‐toxicity, and flexible surface functionalization properties.^[^ ^23^, ^24^ ^]^ AuNPs have also been shown to reduce mortality, renal dysfunction, and liver injury by promoting M1 to M2 polarization.^[^ ^25^, ^26^ ^]^ Our ICAM1‐congugated AuNP nanomedicine successfully delivered the PAD2 inhibitor to M1‐polarized alveolar cells, offering a promising approach for the treatment of ALI.

## 2 Results

### 2.1 Single‐Cell Characterization of *Nlrp3^+^Icam1^+^* Myeloid Cells in Septic Lung

**Figure 1:**
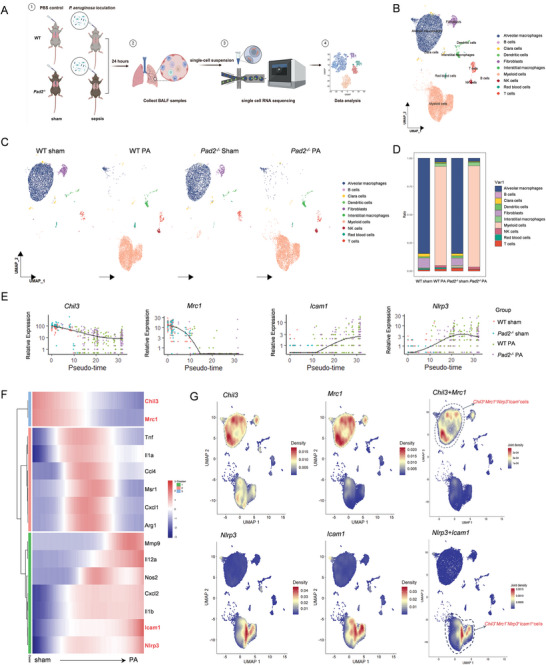
scRNA‐seq analysis of bronchoalveolar lavage fluid (BALF) cells comparing wild‐type and *Pad2^−/−^* mice. A. Schematic diagram showing scRNA‐seq pipeline of murine BALF cells WT and *Pad2^−/−^* mice during PA‐pneumonia induced ALI. BALF cells from 3 mice were pooled into one sample for each group including WT sham, WT PA, *Pad2^−/−^* sham, and *Pad2^−/−^* PA groups. Adapted with permission from BioRender. Yu, X. (2025). B. Nonlinear dimensionality reduction using Uniform Manifold Approximation and Projection (UMAP) analysis of 22193 BALF cells from WT and *Pad2^−/−^* mice reveals ten distinct clusters following unsupervised clustering. Each dot represents an individual cell, with colors indicating cluster assignment. C. Experimental Group‐based UMAP visualization of WT sham, WT PA, *Pad2^−/−^* sham, and *Pad2^−/−^* PA. This visualization highlights the distinct immune landscapes present before and after PA infection, shifting from an alveolar macrophage‐dominant to a myeloid cell‐dominant population. D. Distribution of cell subtype proportions among all cell populations in each experimental group. E. Pseudotime and single‐cell trajectory analysis of *Chil3*, *Mrc1*, *Nlrp3* and *Icam1* genes by Monocle. F. Heatmap of M1/M2 genes, ordered based on their common kinetics through pseudotime using Monocle. G. The upper panel shows expression visualization of *Chil3* and *Mrc1* genes across all cluster populations, along with their co‐expression patterns across distinct cell clusters. The lower panel shows expression of *Nlrp3* and *Icam1* visualization of genes across all cluster populations, along with their co‐expression patterns across distinct cell clusters. Density plots represent the relative gene expression levels.

Using the 10× Genomics scRNA‐seq platform, we analyzed 22193 BALF cells from PA‐induced ALI and sham models in both WT and *Pad2^−/−^* mice (**Figure** **1**A; Figure S1A, Supporting information). Through nonlinear dimensionality reduction on uniform manifold approximation and projection (UMAP) plots, we delineated a diverse immune cell landscape for both WT and *Pad2^−/−^* groups following integration analysis. Ten distinct clusters of BALF cells, including alveolar macrophages (AMs), myeloid cells, fibroblasts, Clara cells, dendritic cells (DCs), T cells, interstitial macrophages (IMs), NK cells, B cells, and red blood cells, were identified using the Seurat clustering algorithm (Figure 1B; Figure S1B, Supporting information). Cell type identification within each cluster relied on the significant expression of well‐characterized marker genes (Figure S1C, Supporting information). UMAP visualization (Figure 1C) and cell ratio analysis (Figure 1D) illustrate the predominance of AMs in the sham condition (84.47% in WT sham group, 84.45% in *Pad2^−/−^* sham group), with myeloid cells emerging as the major population post‐PA infection (87.94% in WT PA group and 89.67% in *Pad2^−/−^* PA group).

To explore the potential relationship between AMs, IMs, and myeloid cells in the sham and PA groups, we analyzed the dynamic immune states and cell transitions within the macrophage/myeloid cell compartment using the Monocle algorithm.^[^ ^27^ ^]^ Pseudotime analysis revealed that AMs from the sham group were predominantly localized at the starting branch of the trajectory, while myeloid cells from the PA group were situated at the terminal end (Figure S1D, Supporting information). IMs, present in both sham and PA conditions, were mainly positioned at the third branch (Figure S1D, Supporting information). Gene expression analysis of macrophages and myeloid cells revealed distinct ALI‐associated phenotypic and functional changes. AMs from the sham group exhibited upregulation of M2‐associated genes, *Chil3* and *Mrc1* (Figure 1E,F). Following PA‐induced ALI, myeloid cells showed increased expression of M1‐related genes, including *Nlrp3, Icam1, Il1b, Cxcl2, Nos2, Il12a* and *Mmp9*,^[^ ^28^ ^]^ along with decreased expression of *Chil3* and *Mrc1*(Figure 1E,F). To further investigate shifts in macrophage polarization between the sham and PA conditions, we evaluated the expression of *Chil3*, *Mrc1*, *Nlrp3* and *Icam1* across all cell populations. Consistent with the pseudotime analysis, *Chil3^+^Mrc1^+^Nlrp3* ^−^ *Icam^−^* cells were primarily AMs from the sham group, whereas *Chil3^−^Mrc1^−^ Nlrp3^+^Icam1^+^* cells were mainly myeloid cells from the PA group (Figure 1G).

### 2.2 *Pad2* Knockout Alters Myeloid Cell Composition and Function in ALI

**Figure 2:**
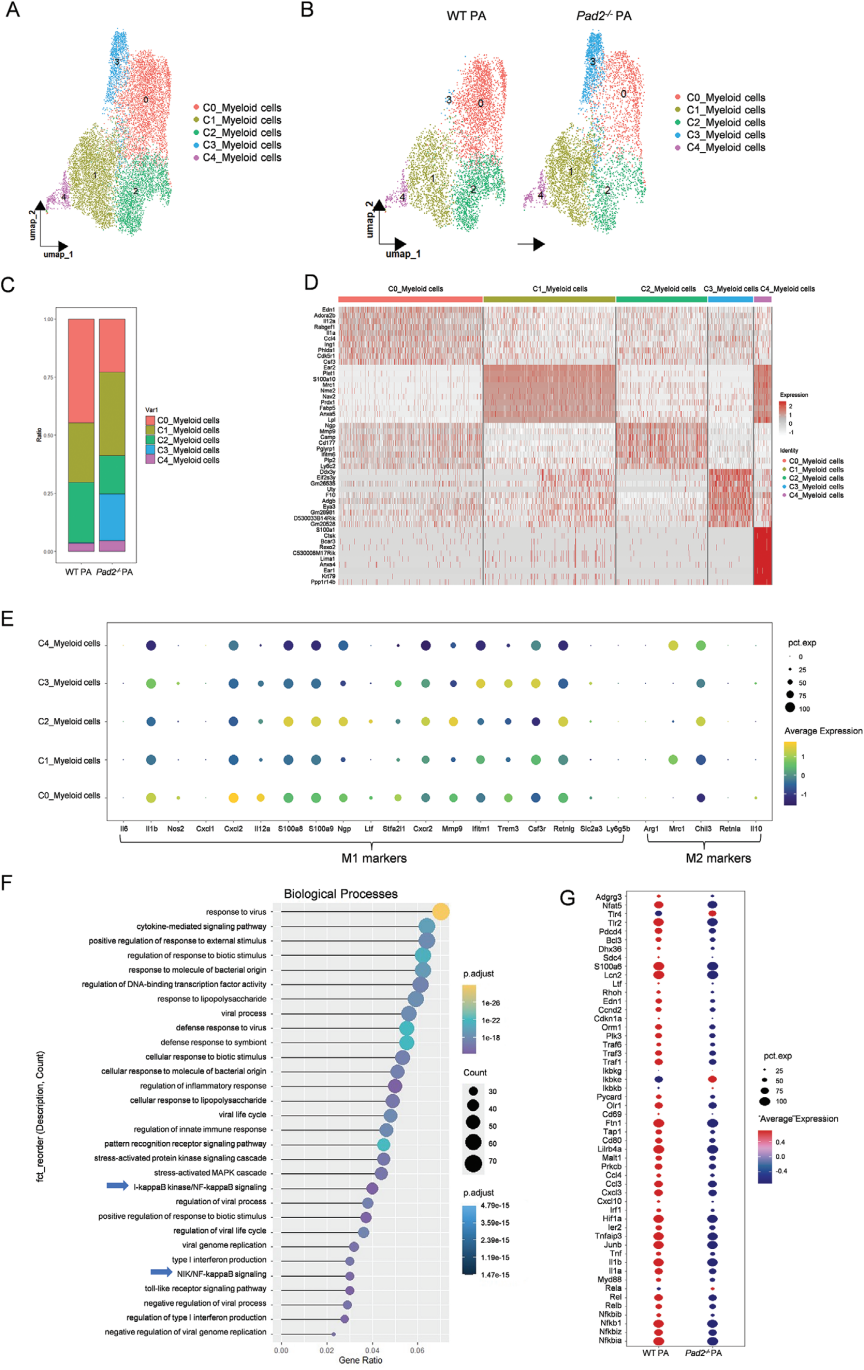
Subcluster analysis of myeloid cells from WT and *Pad2^−/−^* mice after PA‐induced ALI. A. UMAP analysis of five distinct sub‐clusters (Clusters 0, 1, 2, 3, and 4) of myeloid cells from WT and *Pad2^−/−^* PA mice, with each cluster color‐coded for identification. B. Experimental group‐based UMAP visualization of myeloid cells from WT PA and *Pad2^−/−^* PA groups. C. Distribution of cell‐subtype proportions among myeloid cell populations in WT PA and *Pad2^−/−^* PA groups. D. Heatmap showing gene expression within the myeloid cell compartment from WT and *Pad2^−/−^* PA mice in the PA‐induced ALI condition, annotated by cluster types. E. Expression profiles of pro‐inflammatory genes (*Il6, Il1b, Nos2, Cxcl1, Il12a, S100a8, S100a9, Ngp, Ltf, Stfa2l1, Cxcr2, Mmp9, Ifitm1, Trem3, Csf3r, Retnlg, Slc2a3, and Ly6g5b*) and anti‐inflammatory genes (*Arg1, Mrc1, Chil3, Retnla, and Il10*) across myeloid cell clusters, illustrated by dot plots. F. Enrichment analysis of representative GO biological pathways for Cluster 0 myeloid cells, illustrated by dot plots. G. Expression profiles of NF‐κB pathway genes across myeloid cells from WT PA and *Pad2^−/−^* PA groups, illustrated by dot plots.

Given that the *Nlrp3^+^Icam1^+^* myeloid cell compartment represents the largest emergent population of alveolar immune cells after PA‐induced ALI, we performed an unsupervised cluster analysis to investigate the heterogeneity among all myeloid cells. Five transcriptionally distinct subcluster of myeloid cells (Clusters 0–4) were identified (**Figure** **2**A). A heterogeneous cell distribution was observed among WT PA and *Pad2^−/−^* PA groups across these subclusters (Figure 2B,C). Cluster 0 myeloid cells emerged as a prominent population post‐PA infection, characterized by the expression of *Il12a, Il1a, Ccl4*, and *Csf3* genes, indicative of a pro‐inflammatory M1‐like phenotype. Cluster 1 myeloid cells displayed elevated levels of *Mrc1, Anxa5, Ear2*, and *S100a10*, aligning with an M2‐like anti‐inflammatory phenotype. Cluster 2 myeloid cells were characterized by the expression of *Ngp, Mmp9, Camp, Cd177*, and *Ly6c2* genes, suggesting a neutrophil‐like phenotype involved in inflammation and antimicrobial defense. Cluster 3 myeloid cells expressed *Ddx3y, Eif2s3y*, and *Eya3* genes associated with RNA metabolism and protein synthesis in cell development. Finally, Cluster 4 myeloid cells were distinguished by high expression of *S100a1*, *Ctsk*, *Bcar3*, and *Lima1*, which are linked to cell adhesion and migration (Figure 2D).

Next, we compared the differences in the relative proportions of the subclusters among myeloid cells. Notably, the deletion of *Pad2* resulted in a marked reduction of M1‐like Cluster0 myeloid cells (44.7% in WT PA vs 22.9% in *Pad2^−/−^* PA) and an increase in M2‐like Cluster1 myeloid cells (25.5% in WT PA vs 35.8% in *Pad2^−/−^* PA) (Figure 2C). Figure 2E showed the expression of M1/M2‐related genes among all myeloid cell clusters, with Cluster 0 myeloid cells displaying a distinct M1‐polarization signature, characterized by the highest M1‐related genes and the lowest M2‐polarization genes. Gene Ontology (GO) enrichment analysis identified activated pathways in Cluster 0 myeloid cells, suggesting positive regulation of inflammatory responses (Figure 2F). Notably, NF‐κB signaling (NIK/NF‐kappaB signaling and I‐kappaB kinase/NF‐kappaB signaling) was among these activated pathways, confirmed as an important mechanism for promoting M1 polarization state.^[^ ^29^ ^]^ Additionally, GO enrichment analysis for Clusters 1, 2, 3, and 4 is shown in Figure S2A, Supporting information. The heatmap further demonstrates that NF‐κB signaling genes are expressed at higher levels in Cluster 0 myeloid cells compared to other myeloid cell clusters (Figure S2B, Supporting information). Furthermore, the expression of genes critical for NF‐κB signaling was decreased in *Pad2^−/−^* PA compared to WT PA group (Figure 2G).

### 2.3 Effect of PAD2 Inhibition and Knockout on Macrophage Polarization in PA‐Induced ALI

**Figure 3:**
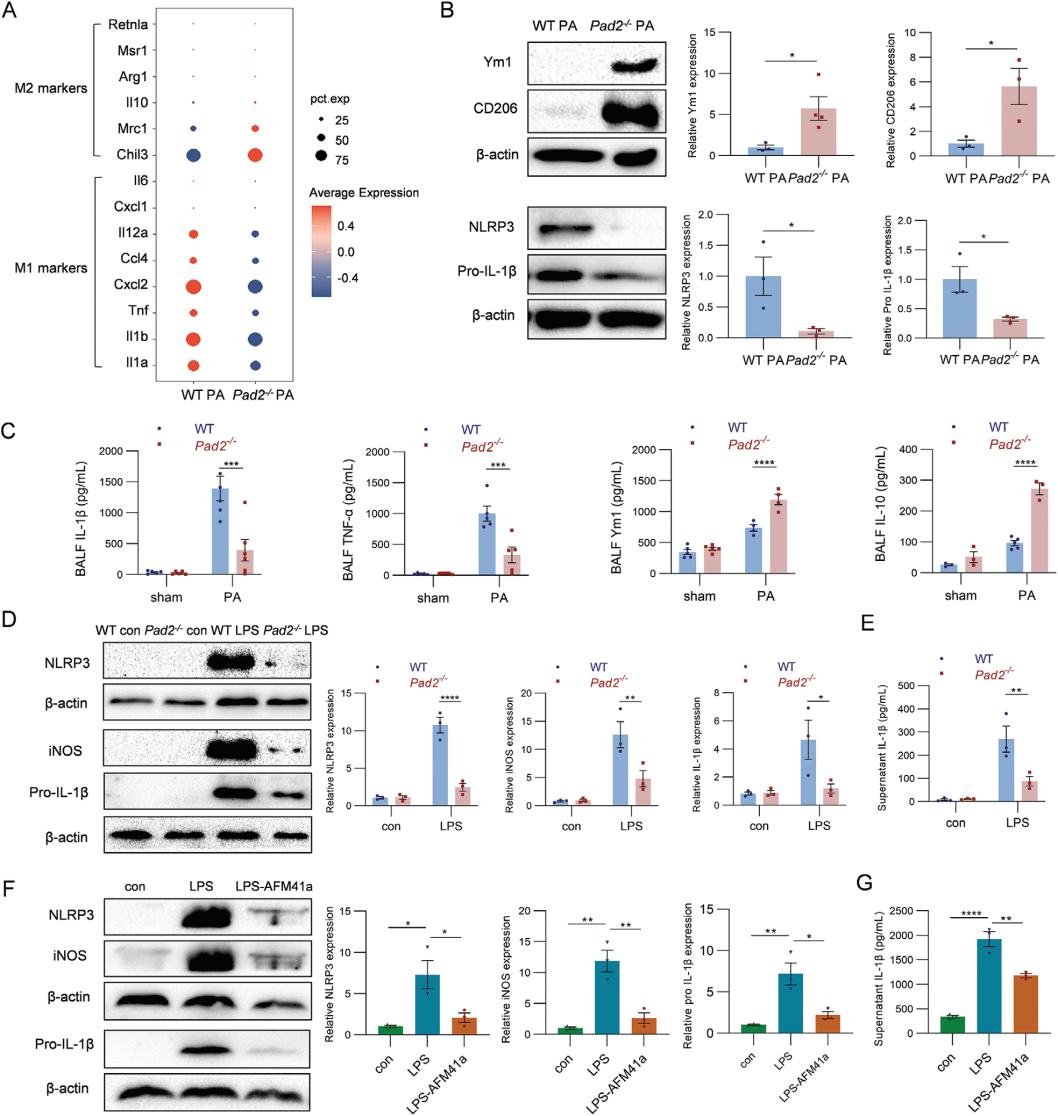
PAD2 modulates M1/M2 polarization in PA‐induced ALI. A. Gene expression levels of M2 markers (*Chil3, Mrc1, Il10, Arg1, Msr1, Retnla*) and M1 markers (*Il1a, Il1b, Tnf, Cxcl1, Cxcl2, Ccl4, Il12a*, *Il6*) across all BALF cells, illustrated in a dot plot for WT PA and *Pad2^−/−^* PA group comparison. B. Western blot analysis for M2 proteins (Ym1 and CD206) and M1 proteins (NLRP3 and pro‐IL‐1β) in BALF cell lysates from WT and *Pad2^−/−^* mice 24 h post‐PA inoculation (*n* = 3). Relative protein expression levels are shown in the right panel. C. ELISA results showing levels of IL‐1β, TNF‐α, Ym1, and IL‐10 in BALF from WT and *Pad2^−/−^* mice, across sham conditions and 24 h post‐PA inoculation (*n* = 3–6). D. Western blot analysis comparing the expression of NLRP3, iNOS, and pro‐IL‐1β proteins in BMDMs from WT and *Pad2^−/−^* mice, treated with PA‐derived LPS (250 ng mL^−1^, 24 h) (*n* = 3). Relative protein expression levels are shown on the right panel. E. ELISA result showing IL‐1β levels in BMDMs culture supernatant from WT and *Pad2^−/−^* mice, treated with PA‐derived LPS (250 ng mL^−1^, 24 h). Cell culture supernatants were analyzed to determine the concentrations of released mature IL‐1β cytokine (*n* = 3). F. Western blot analysis of NLRP3, iNOS, and pro‐IL‐1β proteins in RAW 264.7 cells. Cells were stimulated with 250 ng mL^−1^ PA‐derived LPS or without PA as control, and simultaneously treated with or without 1 µm AFM41a for 24 h (*n* = 3). Relative protein expression levels are shown on the right panel. G. ELISA result showing IL‐1β levels in RAW264.7 cell culture supernatant. Cell culture supernatants were analyzed to determine the concentrations of released mature IL‐1β cytokine (*n* = 3). Results in B–G are representative of at least 3 independent experiments. Data for bar charts in B were analyzed using unpaired Student's *t*‐tests. Data for bar charts in C–E were analyzed using two‐way analysis of variance (ANOVA). Data for bar charts in F and G were analyzed using one‐way ANOVA. Data are presented as means ± SEM. Asterisks (*) denote statistical significance, with *p*‐values indicated as follows: * *p* < 0.05; ** *p* < 0.01; *** *p* < 0.001; **** *p* < 0.0001.

Our scRNA‐seq analysis revealed that myeloid cells exhibited notable plasticity and polarization characteristics in the immune response.^[^ ^30^, ^31^ ^]^ To assess the impact of *Pad2* deletion on M1/M2 polarization function, we conducted a comparative analysis of M1 and M2‐related gene expression across all BALF cells between WT and *Pad2^−/−^* mice. Following PA‐induced ALI, our scRNA‐seq analysis revealed an upregulation of M2‐related genes (*Chil3, Mrc1, Il10, Arg1, Msr1, Retnla*) in *Pad2^−/−^* mice compared to their WT counterparts. Conversely, *Pad2^−/−^* mice showed a downregulation of M1‐related genes (*Il1a, Il1b, Tnf, Cxcl2, Ccl4, Il12a, Cxcl1, Il6*) compared to WT mice after PA infection (**Figure** **3**A).

Subsequent validation through Western blot analysis of BALF cell lysates from WT and *Pad2^−/−^* mice suggested a regulatory role of PAD2 in M1/M2 polarization. Specifically, the *Pad2^−/−^* PA group showed elevated levels of Ym1 and CD206 proteins and lower levels of NLRP3 and IL‐1β proteins (Figure 3B). ELISA assays were conducted to analyze immune biomarkers associated with M1 and M2 phenotypes in BALF from WT and *Pad2^−/−^* mice in both sham and PA groups. Our results revealed the activation of both pro‐inflammatory and anti‐inflammatory markers after PA‐induced ALI (Figure 3C). The levels of M1‐related IL‐1β and TNF‐α were attenuated in *Pad2^−/−^* PA mice, whereas those of M2‐related Ym1 and IL‐10 were significantly higher in this group (Figure 3C). To investigate the influence of *Pad2* deletion in vitro, we treated bone marrow‐derived macrophages (BMDMs) from WT and *Pad2^−/−^* mice with PA‐derived LPS for 24 h. Significant upregulation of M1‐related NLRP3, IL‐1β, and iNOS proteins was observed following LPS treatment, with *Pad2* KO significantly reducing their levels (Figure 3D,E). Furthermore, we used a novel PAD2 inhibitor, AFM41a, to explore whether inhibiting PAD2 activity could affect macrophage polarization.^[^ ^32^ ^]^ We stimulated mouse RAW 264.7 cells with LPS, in the presence or absence of AFM41a, and found that inhibiting PAD2 activity with AFM41a also decreased M1‐polarization protein expression, including NLRP3, IL‐1β and iNOS (Figure 3F,G).

### 2.4 Nuclear Translocation Modulated by PAD2‐Dependent Citrullination of NF‐κB p65

**Figure 4:**
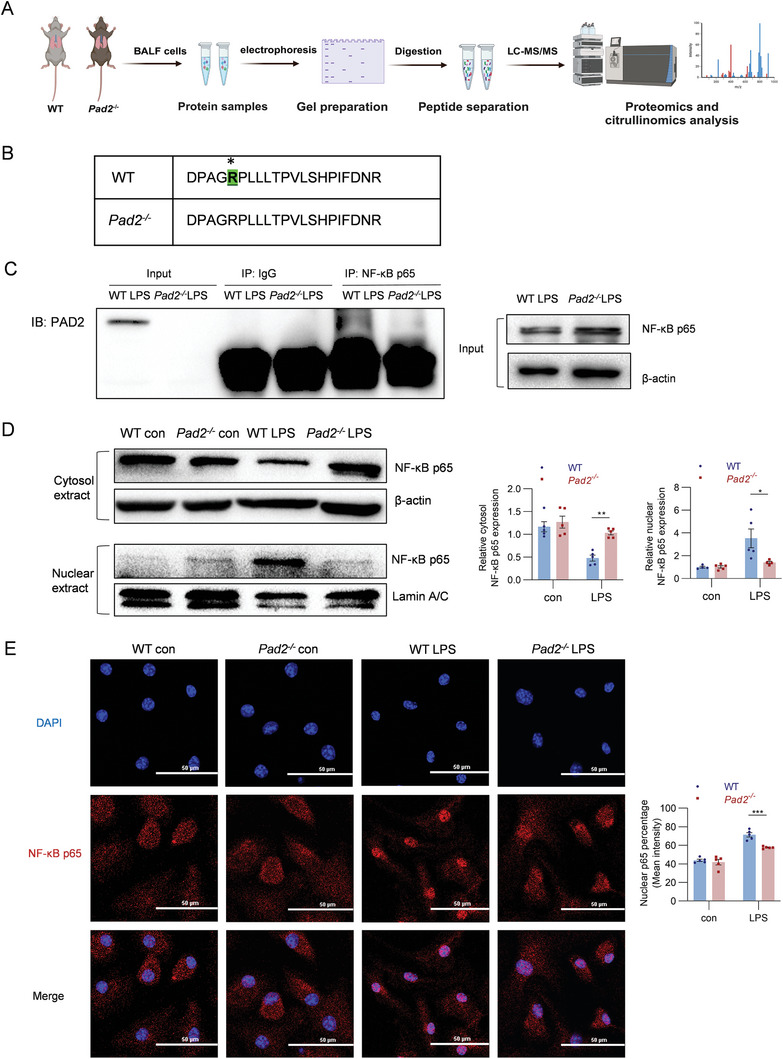
Identification of PAD2‐dependent citrullination site within NF‐κB p65. A. Schematic diagram showing the citrullinome pipeline of murine BALF cells from the WT and *Pad2^−/−^* group. Adapted with permission from BioRender. Yu, X. (2025). B. Mass spectrometry identification of citrullinated peptides at arginine 171 (R171) in NF‐κB p65 from BALF cells of WT mice. The citrullination site is highlighted in green (*). Notably, no citrullinated form of the peptides was identified detected from BALF cells of *Pad2^−/−^* mice, indicating the PAD2‐dependent citrullination at the R171 residue. BALF cells from 3 mice were pooled into one sample. C. BMDMs from WT and *Pad2^−/−^* mice were collected and stimulated with PA‐derived LPS (250 ng mL^−1^, 1 h), and cells were lysed for protein extraction, followed by immunoprecipitation with anti–p65 antibody or control IgG. The immunoprecipitants were probed with anti‐PAD2 by Western blotting. D. BMDMs from WT and *Pad2^−/−^* mice were stimulated with PA‐derived LPS (250 ng mL^−1^, 1 h) and control. Nuclear and cytosol extracts were prepared separately and examined by Western blotting using a p65 antibody, normalized against β‐actin (cytosol extracts) and Lamin A/C (nuclear extracts) (*n* = 4–5). Relative protein expression levels are shown in the right panel. E. Immunofluorescence analysis of NF‐κB p65 expression in BMDMs from WT and *Pad2^−/−^* mice treated with PA‐derived LPS (250 ng mL^−1^, 1 h) compared to control (*n* = 5), with cells stained for NF‐κB p65 (in red) and nuclei counterstained with DAPI (in blue) under a confocal microscope. Relative protein expression levels are shown in the right panel. Scale bars represent 50 µm. Results in C–E are representative of at least 3 independent experiments. Data for all bar charts were analyzed using two‐way ANOVA. Data are presented as means ± SEM. Asterisks (*) denote statistical significance, with *p*‐values indicated as follows: * *p* < 0.05; ** *p* < 0.01; *** *p* < 0.001.

Our investigation demonstrates that PAD2 plays a crucial role in modulating the M1/M2 polarization transition in ALI. NF‐κB is the major transcription factor responsible for activating M1 macrophage polarization.^[^ ^29^ ^]^ Given PAD2's ability to citrullinate proteins and influence their activity, we hypothesized that PAD2 might interact with NF‐κB via citrullination, thereby affecting NF‐κB pathway activity. To test this, we prepared BALF cell lysates from WT and *Pad2^−/−^* mice and subjected them to in‐gel digestion and liquid chromatography‐tandem mass spectrometry (LC‐MS/MS) analysis (**Figure** **4**A). Proteomics and citrullinomics analysis of NF‐κB pathway proteins revealed citrullinated NF‐κB p65, specifically identifying the citrullinated peptide, DPAGRPLLLTPVLSHPIFDNR, which contains R171 (Figure 4B). This citrullinated peptide was detected in the WT group but not in the *Pad2^−/−^* group. The citrullination of R171 was confirmed by observing a ≈1 Da heavier citrullinated peptides in WT mice (2328.28 Da) compared to the uncitrullinated peptides in *Pad2^−/−^* mice (2327.28 Da) in the MS spectrum (Figure S3, Supporting information). To validate the interaction between PAD2 and p65, BMDMs from WT and *Pad2^−/−^* mice were stimulated with PA‐derived LPS for 1 h. Our results showed that anti‐ NF‐κB p65 antibody was co‐immuno‐precipitated PAD2 from the WT group, whereas this co‐immunoprecipitation was inhibited in the *Pad2^−/−^* group (Figure 4C).

Upon external stimuli, cytosolic NF‐κB p65 becomes activated and translocated into the nucleus to regulate the expression of pro‐inflammatory mediators.^[^ ^33^ ^]^ To study this process, we utilized BMDMs from both WT and *Pad2^−/‐^* mice and isolated cytosolic and nuclear extracts separately to assess the levels of NF‐κB p65. Following 1‐h PA‐derived LPS stimulation, we observed decreased expression of cytosolic NF‐κB p65, accompanied by its translocation into the nucleus, resulting in a notable increase in nuclear p65. Conversely, *Pad2* deletion attenuated the accumulation of nuclear NF‐κB p65 while maintaining cytosolic levels after LPS stimulation (Figure 4D). Immunofluorescence analysis of NF‐κB p65 expression in BMDMs from WT and *Pad2^−/−^* mice further confirmed that *Pad2* deletion impairs the nuclear localization of NF‐κB p65 following LPS stimulation (Figure 4E).

### 2.5 Citrullination of NF‐κB p65 at Arginine 171 Enhances its Interaction with Importin α3

**Figure 5:**
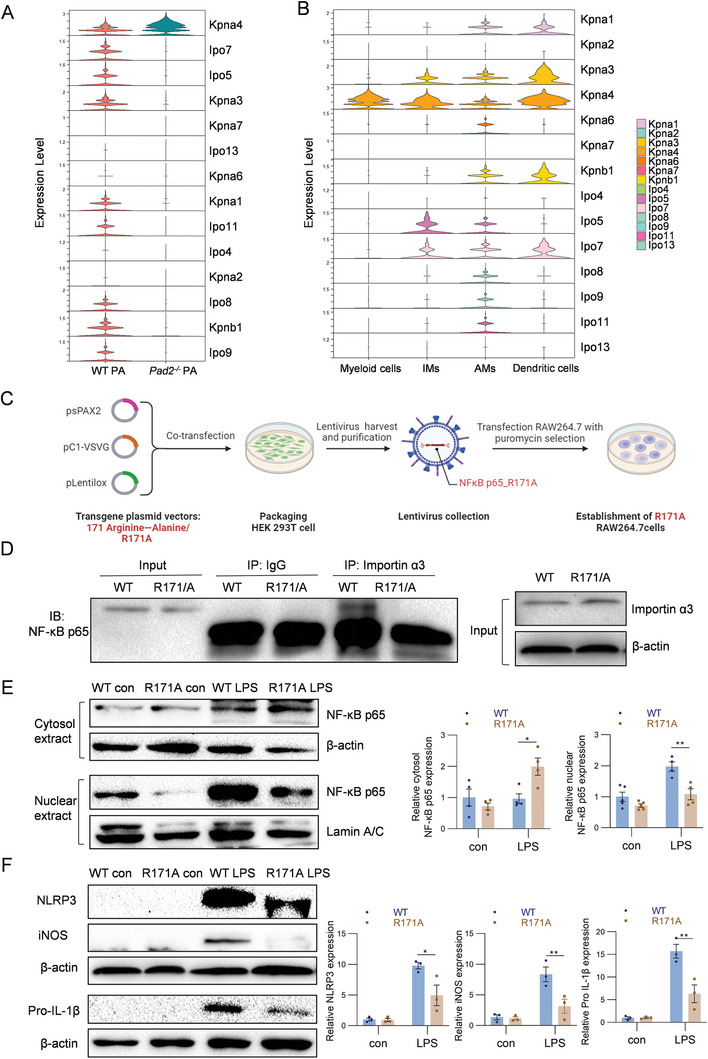
Citrullination of arginine 171 site in NF‐κB p65 modulates its interaction with importin α3. A. Gene expression levels of *Kpna1, Kpna2, Kpna3, Kpna4, Kpna6, Kpna7, Kpnb1, Ipo4, Ipo5, Ipo7, Ipo8, Ipo9, Ipo11* and *Ipo13* across all BALF cells, depicted in a violin plot for WT sham and WT PA group comparison. B. Gene expression level of *Kpna1, Kpna2, Kpna3, Kpna4, Kpna6, Kpna7, Kpnb1, Ipo4, Ipo5, Ipo7, Ipo8, Ipo9, Ipo11* and *Ipo13* across myeloid cells, interstitial macrophages, alveolar macrophages, and dendritic cells, illustrated by violin plots. C. Schematic illustration of the establishment of R171A mutant RAW 264.7 cells using lentivirus transfection. The process involves using a three‐plasmid packaging system to transfect 293T cells, producing and collecting the transgenic lentivirus, and then transducing RAW 264.7 cells. Adapted with permission from BioRender. Yu, X. (2025). D. R171A mutant RAW 264.7 macrophage cells and the corresponding WT cells were stimulated with PA‐derived LPS (250 ng mL^−1^, 1 h), and were lysed for protein extraction, followed by immunoprecipitation with anti–importin α3 or control IgG. The immunoprecipitants were probed with anti‐NF‐κB p65 by Western blotting. E. R171A mutant RAW 264.7 macrophage cells and the WT cells were stimulated with PA‐derived LPS (250 ng mL^−1^, 1 h) and control. Nuclear and cytosol extracts were prepared separately and examined by Western blotting using p65 antibody, normalized against β‐actin and Lamin A/C (*n* = 4–5). Relative protein expression levels are shown in the right panel. F. Western blot analysis of NLRP3, iNOS, and pro‐IL‐1β proteins in R171A mutant RAW 264.7 cells and WT cells, stimulated with PA‐derived LPS (250 ng mL^−1^, 24 h) and control (*n* = 3). Relative protein expression levels are shown in the right panel. Results in D‐F are representative of at least 3 independent experiments. Data for all bar charts were analyzed using two‐way ANOVA. Data are presented as means ± SEM. Asterisks (*) denote statistical significance, with *p*‐values indicated as follows: * *p* < 0.05; ** *p* < 0.01.

NF‐κB p65 remains inactive in the cytoplasm until activated, upon which it is rapidly imported into the nucleus by importin family proteins.^[^ ^34^, ^35^ ^]^ To determine which importin proteins mainly facilitate NF‐κB p65 translocation into the nucleus during PA‐induced ALI, we examined the expression of importin family genes in our scRNA‐seq data. Among BALF cells from WT and *Pad2^−/−^* mice after PA‐induced ALI, only the *Kpna4* gene (encoding importin α3) exhibited significantly elevated expression levels (**Figure** **5**A). Additionally, within our myeloid‐derived cell cluster, including myeloid cells, IMs, AMs, and DCs, *Kpna4* gene displayed notably higher expression compared to other importin isoforms (Figure 5B).

Given our findings that PAD2 citrullinates NF‐κB p65 at the arginine 171 site and regulates its nuclear translocation, we hypothesized that citrullination by PAD2 facilitates its interaction with importin α3, thereby facilitating nuclear translocation efficiency. To test this hypothesis, we generated a stable p65‐R171A mutant RAW 264.7 cell line using plasmids injection and lentivirus transfection (Figure 5C). The R171A mutation replaced the arginine (R) at position 171 with alanine (A), making it incapable of being citrullinated by PAD2 (Figure S4A, Supporting information). Co‐immunoprecipitation experiments confirmed that anti‐importin α3 could co‐immuno‐precipitate NF‐κB p65 in WT cells, whereas this interaction was inhibited in R171A mutant cells (Figure 5D). These findings demonstrate that PAD2‐mediated citrullination of NF‐κB p65 at R171 is crucial for its interaction with importin α3, thereby enhancing nuclear translocation.

To further investigate, we separately isolated cytosolic and nuclear extracts from WT and R171A mutant cells post‐LPS stimulation. Our results demonstrated that the R171A mutant inhibited the translocation of p65 from the cytosol to the nucleus, reducing nuclear translocation efficiency (Figure 5E). Given that NF‐κB p65 activated M1 polarization upon inflammatory stimulation, we treated WT and R171A mutant cells with PA‐derived LPS for 24 h. We observed a significant increase in the expression levels of NF‐κB‐targeted M1 polarization proteins, including NLRP3, IL‐1β, and iNOS, upon LPS stimulation in WT cells. In contrast, the R171A mutant decreased the expression of these M1 polarization proteins (Figure 5F). Additionally, we collected the supernatant from these macrophage cultures and assessed the release of pro‐inflammatory cytokines, including IL‐1β, IL‐6, and TNF‐α, which are also regulated by NF‐κB p65. ELISA results revealed that the R171A mutant reduced the expression of these pro‐inflammatory cytokines after LPS stimulation and inhibited their release from the cells (Figure S4B, Supporting information).

### 2.6 In Vitro Attenuation of Inflammatory Response via Targeted Gold‐Nanoparticle‐Mediated PAD2 Inhibition

**Figure 6:**
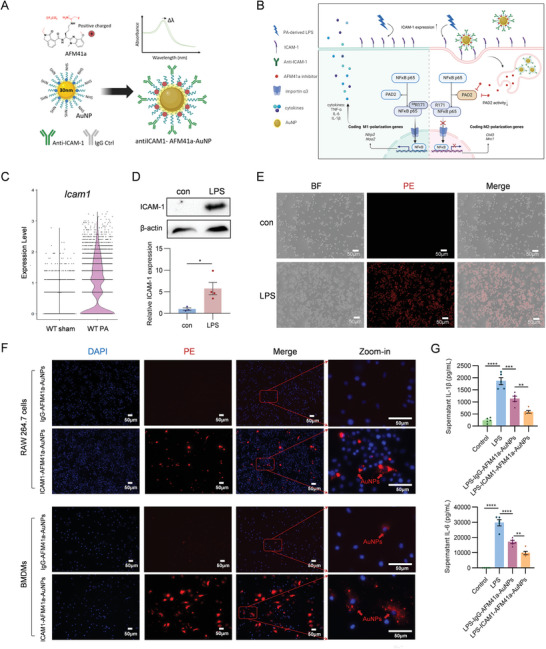
ICAM‐1 antibody‐loaded AuNPs enhance uptake by macrophages and deliver drugs in vitro. A. Schematic illustration of molecular structures of anti‐ICAM1‐AFM41a‐loaded AuNPs and anti‐IgG‐AFM41a‐loaded AuNPs. Adapted with permission from BioRender. Yu, X. (2025). B. Schematic illustration of macrophage polarization state after PA‐derived LPS stimulus and cellular uptake of anti‐ICAM1‐AuNPs. The anti‐ICAM1‐AFM41a‐loaded AuNPs can be taken up by macrophages and directly promote the transition from M1 to M2 polarization. Adapted with permission from BioRender. Yu, X. (2025). C. Violin plots depicting the expression levels of *Icam1* gene in BALF cells from WT sham and WT PA mice. D. Western blot analysis of ICAM‐1 protein in RAW 264.7 cells stimulated with PA‐derived LPS (250 ng mL^−1^, 24 h) and control. Relative ICAM‐1 protein expression level is shown in the right panel (*n* = 3). E. RAW 264.7 cells were stimulated with PA‐derived LPS (250 ng mL^−1^, 6 h) or left untreated as a control. Cells were subjected to immunofluorescence staining with PE anti‐mouse ICAM‐1 antibody to evaluate the expression of ICAM‐1. BF: bright field. Scale bars represent 50 µm. F. RAW 264.7 cells (left panel) and BMDMs (right panel) were pre‐treated with PA‐derived LPS (250 ng mL^−1^, 6 h) or left untreated as control, then treated with PE‐anti‐ICAM1‐AFM41a‐loaded AuNPs or PE‐anti‐IgG‐AFM41a‐loaded AuNPs (20 µg mL^−1^, 3 h). Cells were analyzed by immunofluorescence to assess the uptake of AuNPs. A zoomed‐in view reveals the distribution of AuNPs within the cells. Scale bars represent 100 µm. G. RAW 264.7 cells were pre‐treated with or without PA‐derived LPS (250 ng mL^−1^, 6 h), then treated with PE‐anti‐ICAM‐1‐AFM41a‐loaded AuNPs (20 µg mL^−1^, 18 h). ELISA analyzed cell culture supernatants to show the concentrations of IL‐1β and IL‐6 cytokines (*n* = 5). Results in D‐G were representative of at least three independent experiments. Data for bar charts in D were analyzed using unpaired Student's *t*‐tests. Data for bar charts in G were analyzed using one‐way ANOVA. Data are presented as means ± SEM. Asterisks (*) denote statistical significance, with *p*‐values indicated as follows: * *p* < 0.05; ** *p* < 0.01; *** *p* < 0.001; **** *p* < 0.0001.

To investigate the therapeutic effect of PAD2 on macrophage polarization, we developed a nanodrug delivery strategy involving cell‐specific PAD2 inhibition targeting ICAM‐1, a surface marker highly expressed on M1 macrophages as identified in our scRNA‐seq results (Figure 1E–G). We selected 30 nm gold nanoparticles (AuNPs) (Figure S5, Supporting information) as the drug delivery carrier due to their biocompatibility and non‐toxicity, properties previously demonstrated to mitigate inflammation in mouse models.^[^ ^23^, ^24^ ^]^ A self‐assembled polyethylene glycol (PEG) layer was added to the nanoparticle to passivate the surface and prevent aggregation. These nanoparticles were co‐conjugated with ICAM‐1 antibody or IgG control via a covalent bond, and with a novel PAD2 inhibitor AFM41a through electrostatic interactions (**Figure** **6**A).

To evaluate the loading performance of anti‐ICAM‐1 or control IgG antibodies and the AFM41a inhibitor on the nanoparticles, we used dynamic light scattering (DLS) and zeta potential measurements to determine nanoparticle size and surface charge before and after conjugation. A significant increase in particle size following antibody conjugation confirmed the successful attachment of the larger ICAM‐1 antibody or IgG control via covalent bonding to the AuNP surface (Figure S6A–C). Additionally, the zeta potential shift from mildly negative to mildly positive indicates the successful electrostatic binding of the positively charged small molecule inhibitor AFM41a (Figure S6D). The polydispersity index (PDI) remained ≈0.2, suggesting a narrow size distribution and minimal aggregation. To further quantify the level of antibody loading, we performed a bicinchoninic acid (BCA) assay (Figure S7, Supporting Information), which indicated that ≈93.1 antibody molecules were conjugated per AuNP, confirming efficient surface functionalization. The AFM41a drug loading efficiency was evaluated using high‐performance liquid chromatography (HPLC). A standard curve was established based on the AFM41a peak area, allowing the quantification of the drug loading efficiency by comparing the AFM41a concentrations in the supernatant before and after AuNP conjugation. The HPLC‐based analysis yielded an average AFM41a loading of 7005 molecules per AuNP (Drug/NPs; Figure S8, Supporting Information).

Leveraging ICAM‐1 overexpression in inflamed endothelial cells and macrophages during ALI, the anti‐ICAM‐1 antibody directs AuNP uptake into macrophages via receptor‐mediated endocytosis. Inside the lysosome, high ionic strength and acidic pH (≈4.5–5.0) protonate PEG‐carboxyl groups, weakening electrostatic interactions and facilitating AFM41a release. AuNP accumulation induces osmotic swelling and physical stress (e.g., PEG degradation, aggregation), promoting drug escape into the cytosol, where PAD2 is primarily located. AFM41a inhibits PAD2 activity and the citrullination process. This targeted delivery blocks the PAD2‐NF‐κB p65‐importin α3 pathway, thereby mitigating inflammation and promoting the shift from M1 to M2 polarization (Figure 6B). Following PA‐induced ALI, scRNA‐seq analysis showed a notable upregulation of *Icam1* expression in BALF cells compared to sham mice (Figure 6C). To further validate ICAM1 role in macrophages, RAW 264.7 macrophages were stimulated with PA‐derived LPS, which resulted in a marked increase in ICAM‐1 expression. This finding was confirmed by both Western blot and immunofluorescence results (Figure 6D,E).

To visualize the macrophage uptake of the nanodrug in vitro, we leveraged the localized surface plasmon resonance (LSPR) of the AuNPs to enhance the fluorescence signal of nearby fluorophores. In our assay, individual AuNPs (LSPR peak = 526 nm, Figure S9A, Supporting information) aggregate within the macrophage cytoplasm after being engulfed. We selected phycoerythrin (PE) fluorophores with an emission wavelength matching the LSPR peak wavelength of the aggregated AuNPs to facilitate plasmon‐enhanced fluorescence (PEF).^[^ ^36^ ^]^ Both the ICAM1‐AFM41a‐AuNPs (nanodrug conjugates) and their non‐targeted IgG‐AFM41a‐AuNPs controls were tagged with a secondary PE‐anti‐Rat‐IgG antibody complex, and we confirmed their strongly enhanced fluorescence intensity upon aggregation prior to application (Figure S9B, Supporting information).

After LPS stimulation, BMDMs were incubated with ICAM1‐AFM41a‐AuNPs at concentrations of 1, 5, 10, 20, and 40 µg mL^−1^ (nanodrug‐treated group), or without ICAM1‐AFM41a‐AuNPs (untreated group). Both groups were imaged after 3 h (see Experimental Section). We observed a remarkable granulate‐like fluorescence signal (indicating AuNP aggregates) in the cytoplasm of the nanodrug‐treated BMDMs, with the signal increasing proportionally with the dosing concentration of the ICAM1‐AFM41a‐AuNPs nanodrug (Figure S9C, Supporting information). AuNP uptake was also evaluated between the ICAM‐1 target group (ICAM1‐AFM41a‐AuNPs) and the IgG control group (IgG‐AFM41a‐AuNPs) with LPS stimulation, to assess M1‐targeted delivery in macrophages. The anti‐ICAM‐1 target group showed a notably stronger uptake compared to the IgG control group, in both RAW264.7 cells and BMDMs (Figure 6F). To assess the anti‐inflammatory effect of the ICAM1‐AFM41a‐AuNPs, we collected supernatant from each group 24 h after LPS stimulation. Treatment with AuNPs led to a marked reduction in the levels of pro‐inflammatory cytokines IL‐1β and IL‐6 in both the anti‐ICAM1 target and IgG control groups compared to the untreated group. Importantly, the anti‐ICAM1 target group exhibited a significantly greater reduction in IL‐1β and IL‐6 production compared to the IgG control group (Figure 6G).

### 2.7 Therapeutic Efficacy of ICAM1‐AFM41a‐AuNPs in PA‐Induced ALI

**Figure 7:**
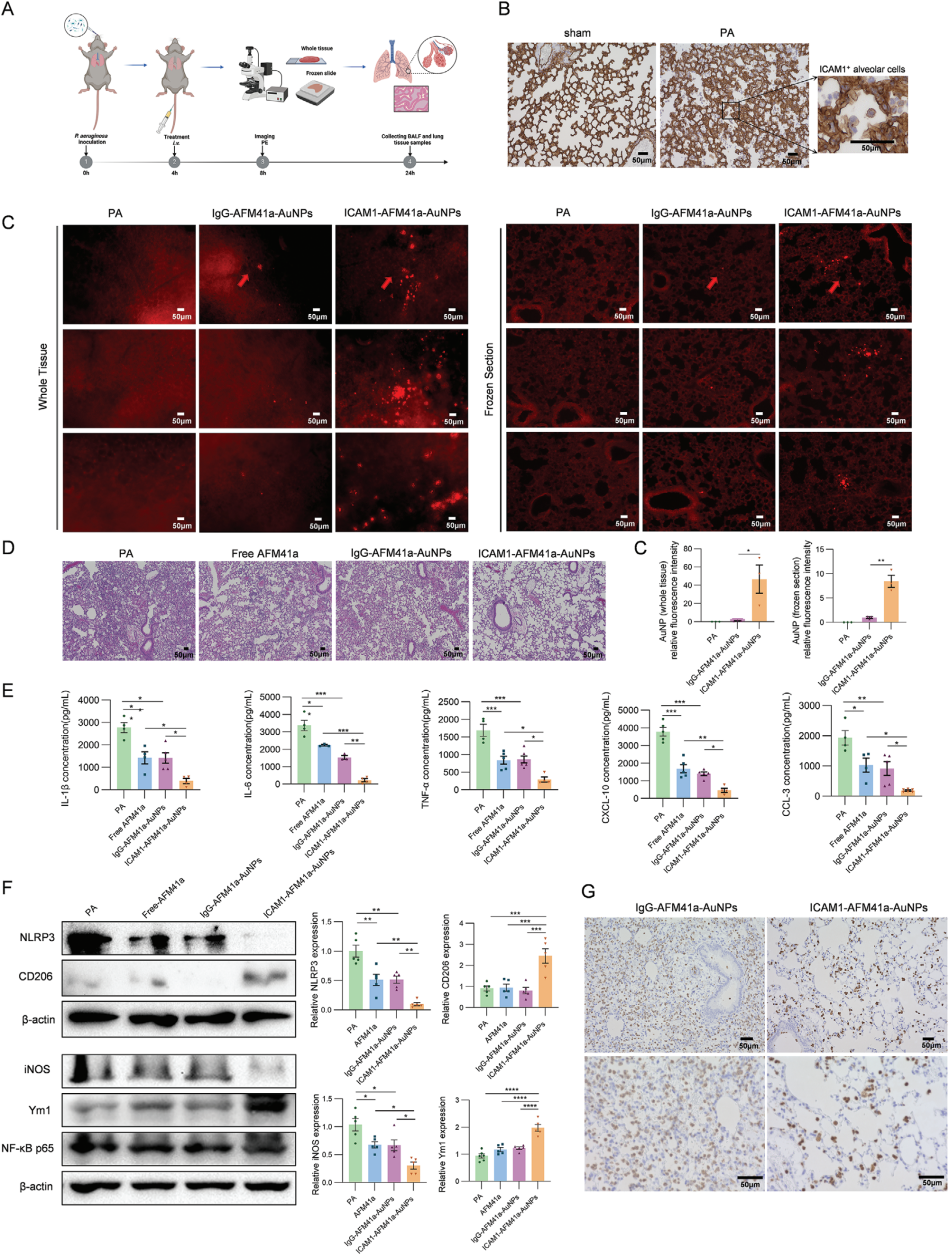
Therapeutic performance of the ICAM‐1 antibody‐loaded AuNPs in PA‐induced ALI. A. Experimental procedures for evaluating the targeting and therapeutic efficacy of AuNPs in PA‐induced ALI. 4 h after PA inoculation, free AFM41a, PE‐anti‐IgG‐AFM41a‐loaded AuNPs, and PE‐anti‐ICAM1‐AFM41a‐loaded AuNPs were administered intravenously. Lungs were collected for imaging 8 h post‐infection. Blood, BALF, and lung tissue were collected 24 h post‐infection. Adapted with permission from BioRender. Yu, X. (2025). B. Immunohistochemical (IHC) staining for ICAM‐1 in lung tissues of sham and PA‐induced ALI mice. A zoomed‐in view shows ICAM1^+^ alveolar myeloid cell from PA group. Scale bars represent 50 µm. C. The immunofluorescence of PE‐labeled anti‐IgG AuNPs and anti‐ICAM1‐AFM41a‐loaded AuNPs were measured using whole lung tissue (left panel) and lung frozen sections (right panel) to analyze lung‐targeted efficacy of AuNPs. The red arrows indicate the locations of AuNPs. Scale bars represent 50 µm. The statistical analysis results of fluorescence intensity are shown in the lower right panel. D. Histopathological examination of lung injury using H&E‐stained lung tissue sections from mice with free AFM41a, PE‐anti‐IgG‐AFM41a‐loaded AuNPs, PE‐anti‐ICAM1‐AFM41a‐loaded AuNPs, or without treatment 24 h after PA infection. Scale bars represent 50 µm. E. ELISA results showing concentrations of IL‐1β, IL‐6, TNF‐α, CXCL‐10, and CCL‐3 in the BALF of mice treated with free AFM41a, PE‐anti‐IgG‐AFM41a‐loaded AuNPs, PE‐anti‐ICAM1‐AFM41a‐loaded AuNPs, or without treatment mice 24 h post‐PA infection (*n* = 3–6). F. Western blot analysis for NLRP3, CD206, iNOS, Ym1 and NF κB p65 proteins in BALF cell lysates from mice treated with free AFM41a, PE‐anti‐IgG‐AFM41a‐loaded AuNPs, PE‐anti‐ICAM1‐AFM41a‐loaded AuNPs, or without treatment mice 24 h post‐PA infection (*n* = 5). Relative protein expression levels are shown in the right panel. G. IHC staining for Ym1 in lung tissues of mice treated with PE‐anti‐IgG‐AFM41a‐loaded AuNPs or PE‐anti‐ICAM1‐AFM41a‐loaded AuNPs within the PA‐induced ALI group (*n* = 3). Scale bars represent 50 µm. Results in B‐G are representative of at least three independent experiments. Data for bar charts in C were analyzed using unpaired Student's *t*‐tests. Data for bar charts in E and F were analyzed using one‐way ANOVA. Data are presented as means ± SEM. Asterisks (*) denote statistical significance, with *p*‐values indicated as follows: * *p* < 0.05; ** *p* < 0.01; *** *p* < 0.001, **** *p* < 0.0001.

To evaluate the targeted delivery and therapeutic efficacy of ICAM1‐AFM41a‐AuNPs nanodrug in a PA‐induced ALI model, we conducted an in vivo experiment as outlined in **Figure** **7**A. Immunohistochemical (IHC) staining was performed to assess ICAM‐1 expression in lung tissues from both sham and PA‐treated mice. ICAM‐1 was primarily expressed in pulmonary vascular endothelial cells under sham conditions, whereas its expression was upregulated in activated alveolar myeloid cells following PA‐induced ALI (Figure 7B). Additionally, IHC staining for ICAM‐1 was performed on the heart, liver, spleen, and kidney in PA‐induced ALI mice. Notably, ICAM‐1 expression was higher in the lung compared to other organs, supporting the targeted delivery of anti‐ICAM1‐Ab‐conjugated‐AuNPs to lung tissue (Figure S10, Supporting information). To further investigate the effect of AuNPs in vivo, mice were inoculated with PA to induce ALI, followed by intravenous administration of ICAM1‐AFM41a‐AuNPs, IgG‐AFM41a‐AuNPs, and free AFM41a 4 h after PA infection. Lungs were harvested for microscopic imaging 8 h after PA infection. We visualized nanoparticle distribution within the whole lung tissue and within frozen sections of the lung through plasmon‐enhanced fluorescence. The intensity of the enhanced fluorescence was sufficient to mask the autofluorescence background of the lung tissue. The frozen sections show that the ICAM1‐AFM41a‐AuNPs nanodrug could transport from the bloodstream to the alveolar airspace, where most macrophages and PA bacteria are located (Figure 7C). Multiple sites within the lung tissue showed significantly higher accumulation and stronger fluorescence emission in ICAM1‐AFM41a‐AuNPs‐treated mice compared to IgG‐AFM41a‐AuNPs‐treated controls, demonstrating that the ICAM1‐AFM41a‐AuNPs nanodrug specifically targeted the injured lung areas.

Further analysis included histological and cytokine assessments of the lung tissues and BALF 24 h after infection. Histological examination revealed a significant reduction in inflammatory cell infiltration, bacterial spread, and cellular debris in the ICAM1‐AFM41a‐AuNPs‐treated mice compared to both IgG‐AFM41a‐AuNPs‐treated and free AFM41a‐treated mice, indicating enhanced therapeutic efficacy of ICAM1‐AFM41a‐AuNPs (Figure 7D). Cytokine analysis demonstrated decreased levels of pro‐inflammatory markers: IL‐1β, IL‐6, TNF‐α, CXCL‐10, and CCL‐3 in the BALF from ICAM1‐AFM41a‐AuNPs‐treated mice, affirming the anti‐inflammatory effect of the nanodrug (Figure 7E). BALF cells were further analyzed for the expression of M1 markers (NLRP3, iNOS) and M2 markers (CD206, Ym1) by Western blot. As shown in Figure 7F, treatment with free AFM41a and IgG‐AFM41a‐AuNPs reduced NLRP3 and iNOS expression but did not significantly affect CD206 and Ym1 levels after PA infection. In contrast, treatment with ICAM1‐AFM41a‐AuNPs significantly decreased NLRP3 and iNOS expression while increasing CD206 and Ym1 expression. Additionally, total NF‐κB p65 protein levels remain unchanged across all groups (Figure 7F). IHC staining of Ym1 confirmed that ICAM1‐AFM41a‐AuNPs treatment increased Ym1 expression in alveolar cells, indicating enhanced M2 polarization (Figure 7G).

## 3 Discussion

The composition and function of alveolar immune cells provide insights into the pathogenesis of ALI.^[^ ^6^, ^37^ ^]^ Our analysis of single‐cell transcriptional profiles of alveolar immune cells reveals significant transitions in macrophage polarization phenotypes in PA‐induced ALI. Specifically, we observed co‐activation of *Nlrp3^+^Icam1^+^* M1‐like cells during PA‐induced ALI, replacing the M2‐like cells present in unstimulated alveoli (Figure 1E–G). This finding is supported by recent studies suggesting that NLRP3 is a key regulator of macrophage‐induced inflammation and M1 polarization.^[^ ^38^, ^39^ ^]^ ICAM‐1 is well‐known for its role in regulating leukocyte adhesion and recruitment from circulation to sites of inflammation in vascular endothelial cells. However, ICAM‐1 expression is also induced in macrophages upon inflammatory stimulation, and its function and mechanisms in macrophages are less well understood.^[^ ^40^, ^41^ ^]^ Our data demonstrate that ICAM‐1 is notably upregulated on macrophages both in vivo and in vitro upon proinflammatory stimulus (Figures 1, 6). Several recent studies have implicated ICAM‐1 in the regulation of macrophage polarization.^[^ ^28^, ^42^, ^43^ ^]^ In line with these studies, our findings suggest that the activation of *Nlrp3^+^Icam1^+^* myeloid cells contributes to M1 polarization and exacerbated inflammation in the pathobiology of PA‐induced ALI.

Subcluster analysis of *Nlrp3^+^Icam1^+^* myeloid cells revealed that C0_myleoid cells exhibited an M1‐polarization state and activated NF‐κB signaling (Figure 2E,F). The NF‐κB signaling pathway, predominantly composed of the p50/p65 heterodimer, mediates the transcriptional expression of NLRP3, ICAM‐1, and proinflammatory cytokines, promoting M1 polarization.^[^ ^44^, ^45^, ^46^ ^]^ Additionally, we observed a shift from M1 to M2 polarization phenotype following *Pad2* deficiency (Figure 3A). Our study indicates that both *Pad2* gene knockout and PAD2 inhibition can mitigate excessive M1 polarization‐associated inflammation in vivo and in vitro (Figure 3B–G). *Pad2* deletion attenuated the activity of the NF‐κB pathway in ALI (Figure 2G). Previous studies have demonstrated that decreased NF‐κB activity can contribute to an M2 polarization shift and reduce lung injury.^[^ ^47^, ^48^, ^49^ ^]^ Based on these findings, we hypothesized that PAD2 might directly citrullinated NF‐κB proteins, thereby promoting M1 polarization and inflammation. Through MS‐based proteomics analysis of alveolar cells, we identified PAD2‐dependent citrullination of NF‐κB p65 at arginine 171 (Figure 4B; Figure S3, Supporting information). The reduced translocation of cytosolic p65 into the nucleus due to *Pad2* deficiency after PA further validated our hypothesis (Figure 4A–C).

To explore PAD2's role in citrullinating the R171 site, we generated R171A mutant RAW 264.7 cells alongside WT controls. Notably, mutations at the R171 site reduced p65's interaction with importin α3 (Figure 5D), impairing its nuclear translocation efficiency in LPS‐stimulated macrophages (Figure 5E). These findings demonstrate that PAD2‐mediated citrullination at R171 is crucial for the downstream interaction between p65 and importin α3, thereby facilitating p65's nuclear localization. Previous studies have shown that PAD4 can directly citrullinate p65 at residues R35, R50, R73, and R149 in neutrophils, activating NF‐κB and prolonging inflammation in vitro.^[^ ^44^ ^]^ In contrast, our study reveals the critical role of PAD2 in citrullinating p65 at R171 in macrophages, influencing their polarization. Notably, PAD2 has been shown to significantly improve survival rates in various sepsis‐induced ALI models compared to PAD4.^[^ ^11^, ^12^ ^]^ Our study contributes to understanding the underlying mechanisms of PAD2's role in ALI immunology, particularly through in vivo studies.

Our study supports the idea that shifting M1‐polarized cells toward anti‐inflammatory M2 polarization in alveoli through targeted delivery of PAD2 inhibitor‐AFM41a can alleviate PA‐induced ALI. Conventional therapeutics for intravenous small‐molecule AFM41a inhibitors face major drawbacks, including systemic distribution that leads to insufficient drug concentrations at the site of lung injury and a lack of cell‐specific targeting ability.^[^ ^19^, ^50^ ^]^ To address these issues, we demonstrated that ICAM‐1 antibody‐loaded AuNPs can specifically deliver the AFM41a inhibitor to *Icam1^+^* M1‐polarized cells in the injured alveoli. During PA‐induced ALI, both activated alveolar cells and lung vasculature highly express ICAM‐1 (Figure 7B; Figure S10, Supporting information),^[^ ^21^, ^22^ ^]^ making it an effective mediator for transporting AuNPs from the bloodstream to the lung airspace. Once in the alveoli, the ICAM‐1 antibody targets *Icam1^+^* M1‐polarized cells, allowing AFM41a to inhibit PAD2's citrullination activity and the associated PAD2‐NF‐κB p65‐importin α3 pathway. Our result showed that ICAM‐1‐coated AuNPs significantly increased enhanced the delivery of AFM41a into the injured alveoli compared to the IgG control (Figure 7C).

Moreover, previous studies have shown that AuNPs can attenuate inflammation and promote M2 polarization,^[^ ^25^, ^26^ ^]^ making them a dual‐function M2‐polarizing nanomedicine when combined with the novel AFM41a inhibitor. Our study demonstrated that ICAM1‐AFM41a‐AuNPs efficiently target M1‐polarized alveolar cells and induce a notable M1‐to‐M2 polarization shift in PA‐induced ALI, compared to non‐targeted AuNPs and free AFM41a treatment (Figure 7E–G). Therefore, this specific M1‐like macrophage‐targeted AuNP therapy holds promise for mitigating the hyperinflammatory response in ALI. However, its efficacy in human ALI remains uncertain, and the safety and effectiveness of ICAM1‐AFM41a‐AuNPs must be validated in clinical trials. Additionally, due to the low abundance of citrullination events in whole‐cell extracts,^[^ ^51^, ^52^, ^53^ ^]^ we identified only one citrullination site at R171 by comparing data from WT and *Pad2^−/−^* mice. It remains unclear whether other arginine residues are also citrullinated by PAD2 and whether R171 is the most critical site. Further investigation into all potential citrullination sites on p65 is necessary and could reveal additional therapeutic targets.

In summary, our scRNA‐seq analysis reveals that PA‐induced ALI activates the NF‐κB pathway in alveolar cells, creating a persistent M1‐like pro‐inflammatory microenvironment. PAD2‐mediated citrullination of NF‐κB p65 at arginine 171 enhances its interaction with importin α3, facilitating nuclear localization and promoting NF‐κB‐mediated M1 polarization. Importantly, our findings demonstrate that targeting M1‐polarized macrophages with ICAM‐1 antibody‐loaded AuNPs delivering the novel AFM41a inhibitor, effectively shifts M1 macrophages toward an M2 phenotype. This approach offers a promising therapeutic strategy for ALI.

## 4 Experimental Section

### Animals

Wild‐type male C57BL/6J and *Pad2^−/−^* mice (8–12 weeks old) were used in this study. Wild‐type mice were purchased from the Jackson Laboratory (Bar Harbor, ME, USA). *Pad2^−/−^* mice were kindly provided by Dr Scott Coonrod (Cornell University, Ithaca, New York, USA). The mice were housed in a specific pathogen‐free environment with a controlled temperature of 23 ± 1.5 °C and relative humidity of 70 ± 20%. All mice used for in vivo and in vitro experiments were age‐ and sex‐matched.

All animal experiments were performed with approval from the University of Michigan (PRO00011567).

### Acute Lung Injury Model

Mice aged 8–12 weeks were exposed to intranasal administration of PA (19660; ATCC) solution to induce PA‐induced ALI. Briefly, a PA solution was prepared at a concentration of 8.25 × 10^7^ colony‐forming units (CFU) mL^−1^ in PBS (Gibco). The mice were anesthetized with ketamine (Dechra Veterinary Products, 80 mg kg^−1^) and xylazine (Akorn, 20 mg kg^−1^), and then held vertically. Subsequently, PA solution (15 µL) was instilled into each nostril, totaling 30 µL to achieve a final bacterial load of 2.5 × 10^6^ CFU. Mice inoculated with sterile PBS (30 µL) served as sham controls. All mice were euthanized by CO_2_ 24 h after inoculation.

### Sample Preparation and Single‐Cell RNA Sequencing

BALF samples were collected from both WT and *Pad2^−/−^* mice after PA infection and sham control. BALF samples from three mice per group were pooled to generate samples (*n* = 3 mice per sample) for the isolation of BALF cells. The BALF samples were centrifuged for 5 min at 400 rcf at 4 °C. The cell pellet was then resuspended in red blood cell lysis buffer (500 µL, 00‐4333‐57; eBioscience) and incubated for 5 min at room temperature. Following this, cold PBS (500 µL, Gibco) was added to dilute the RBC lysis buffer, and the mixture was centrifuged for 5 min at 400 rcf at 4 °C. The resulting BALF cells were resuspended in PBS containing 1% weight/volume FBS (Gibco), and cell viability was determined using automated cell counters (Invitrogen). The single‐cell suspension was thoroughly mixed and loaded onto a 10× Chromium system to capture no more than 10 000 single cells using the Chromium Next GEM Single Cell 3′ GEM, Library & Gel Bead Kit (10× Genomics). The cells were partitioned into Gel Beads in the Chromium instrument. DNA amplification and library construction were performed with cell lysis and barcoded reverse transcription of RNA. The resulting libraries were sequenced using an Illumina HiSeq 4000 next‐generation sequencing platform. Data quality analysis and mapping to ensemble gene symbols were conducted using CellRanger (10x Genomics).

### scRNA‐Seq Data Processing

The CellRanger output data were imported into the Seurat R package (version 5.0.1) for unsupervised clustering analysis. Prior to clustering, filtering procedures were implemented to eliminate multiplets and damaged cells, while sources of variation deemed uninformative were regressed out. Identification of variable genes was achieved through iterative selection based on the dispersion versus average expression profile of each gene. Normalization and scaling of gene expression values within individual cells were conducted using the SCTransform algorithm. Dimensionality reduction and visualization of the data were performed using Principal Components Analysis^[^ ^54^ ^]^ and Uniform Manifold Approximation and Projection (UMAP), incorporating the top 30 principal components. Parameters for UMAP were set to min. dist. = 0.3 and n. neighbor = 30. Subsequently, cells were clustered using an unsupervised clustering approach with default parameters for the Seurat package (resolution = 0.6). Cluster‐specific marker genes were identified utilizing the FindAllMarkers function in Seurat, with criteria set at a *p*‐value < 0.01 and log (fold change) > 0.25 within the target cluster. Visualization of gene expression patterns across cell clusters was accomplished using UMAP plots and heatmaps generated with functions available in the Seurat package. Differentially expressed genes (DEGs) were determined using the FindAllMarkers function with default parameters, specifying log (fold change) > 0.26, *p*‐value < 0.01, and min.pct > 0.1. For Gene Ontology (GO) enrichment analysis, DEGs across different cell types were utilized to conduct cluster‐specific pathway enrichment analysis. Myeloid cell re‐clustering, UMAP visualization, DEG analysis, and GO enrichment analysis were performed with dims = 1:15, resolution = 0.2, and the same default parameters as previously described.

For single‐cell trajectory analysis, Monocle v.2.30.0 was utilized to explore developmental trajectories among alveolar macrophages, interstitial macrophages, and myeloid cells.^[^ ^27^, ^55^ ^]^ The scRNA‐seq data were scaled, normalized, and clustered in Seurat, then downsampled to 1500 cells and imported into a Monocle object. DEGs for each cluster were identified using the FindVariableFeatures function, and the top 2000 genes with the lowest *q*‐values were selected for pseudotime analysis. Cell trajectories were visualized using the plot_cell_trajectory function, with the trajectory's starting point (root_state) defined by cells from the sham condition. Plot_genes_in_pseudotime function and plot_pseudotime_heatmap function were used to show the dynamic trend of the selected significant gene expression levels.

### Cell Culture

Bone marrow‐derived macrophages (BMDMs) were isolated from tibiae and femurs of both WT and *Pad2^−/−^* mice for in vitro experiments. Bone marrow cells were harvested and cultured in 75 mm^2^ Petri dishes containing IMDM (Gibco) supplemented with 20% FBS, 1% penicillin/streptomycin (Lonza Inc), and 30% L929 cell (CCL‐1; ATCC) supernatant at 37 °C/5% CO_2_ incubator. The L929 cell supernatant was generated by incubating L929 cell fibroblasts in IMDM with 10% FBS for 6 days to produce macrophage colony‐stimulating factor (M‐CSF). After 7 days, BMDMs were collected and resuspended in Opti‐MEM (Gibco) to the desired concentrations. Mouse RAW 264.7 cells were cultured in DMEM (Gibco) supplemented with 10% FBS and 1% penicillin/streptomycin at 37 °C/5% CO_2_ incubator. For experiments involving LPS stimulation, cells were challenged by PA‐deprived LPS (250 ng/mL, L9143; Sigma–Aldrich) in Opti‐MEM, while control cells were treated with Opti‐MEM alone for the same duration. For experiments assessing AFM41a inhibitor treatment, cells were exposed to PA‐deprived LPS (250 ng mL^−1^) with or without AFM41a (1 µm) inhibitor followed by a 24‐h incubation.

### Preparation of AuNPs

Custom‐synthesized 30 nm spherical gold nanoparticles functionalized with NHS‐PEG‐SH were ordered from Nanopartz (DC11‐30). The surface charge was adjusted to a desired range of −25 to −30 mV to enable electrostatic binding with the positively charged AFM41a inhibitor. A 2.6 mg quantity of NHS‐PEG‐terminated AuNPs was first dissolved in PBS (0.5 mL) and sonicated for 1 min to form a uniform colloidal solution. Next, sterilized low endotoxin, azide‐free (LEAF) mouse anti‐ICAM‐1 antibody (200 µg, 116132; BioLegend) or rat IgG2b control (200 µg, 408210; BioLegend), along with AFM41a inhibitor (10 µg), were added to create a total solution volume of 1 mL. The mixture was vortexed and incubated for 30 min at 30 °C, followed by centrifugation (12 000 rcf, 5 min). The supernatant was removed, and the pellet was resuspended in 0.01× PBS with 0.1% Tween 20. This washing step was repeated three times. After the final centrifugation, the pellet was resuspended in 1x PBS and stored at 4 °C. For imaging study, the ICAM‐1 or IgG conjugated AuNPs were then stained with PE‐anti‐rat IgG2b antibody (4 µg mL^−1^, 408213; BioLegend) for 30 min at room temperature (25 °C) and subjected to the same washing protocol described above.

### Binding of AuNPs to Macrophages In Vitro

BMDMs and RAW 264.7 cells were seeded in culture slides (354108; Corning) and incubated overnight. The cells were then treated with or without PA‐derived LPS (250 ng mL^−1^) for 6 h, followed by three washes with PBS. Cells were subsequently incubated with PE‐fluorescent labeled ICAM‐1Abs‐coated AFM41a‐loaded AuNPs or PE‐fluorescent labeled IgG2b Abs‐coated AFM41a‐loaded AuNPs sample at 37 °C/5% CO_2_ incubator. For imaging, after 3 h of incubation with AuNPs, cells were washed three times with 1x PBS, and fixed with 4% paraformaldehyde for 30 min at room temperature. Prolong Gold anti‐fade reagent with DAPI (P36931; Invitrogen) was added to the cells, and a coverslip was applied. The cells were imaged by a KEYENCE BZ‐X800 microscope. For cytokines analysis, after 18 h of incubation with AuNPs, cells were washed three times with PBS, and the culture supernatants were collected by centrifugation (300 rcf, 5 min) for subsequent analysis.

### In Vivo Delivery of AuNPs

An ALI mouse model was generated as previously described. 4 h post‐PA inoculation, the mice were grouped randomly and intravenously (i.v.) with either PE‐fluorescent labeled ICAM‐1 Abs‐coated AFM41a‐loaded AuNPs (1 mg per kg of body weight), PE‐fluorescent labeled IgG2b Abs‐coated AFM41a‐loaded AuNPs (1 mg kg^−1^) or free AFM41a (20 mg kg^−1^), or PBS as a control. At predetermined time intervals, lungs were collected at 4 h post i.v. and both lung tissue and frozen lung sections (10 µm) were prepared for imaging using KEYENCE BZ‐X800 microscope. 24 h post‐PA inoculation, the mice were euthanized, and BALF and lungs were collected for further analysis.

### Measurement of Cytokines and Chemokines

The levels of IL‐1β, TNF‐α, IL‐10, Ym1, IL‐6, CXCL‐10, and CCL‐3 in BALF and supernatant were measured by the core of UMICH Immune Monitoring Shared Resource using the core‐developed sandwich ELISA.

### Western Blotting

For Western blotting, cells were washed two times with ice‐cold PBS and lysed with RIPA buffer (89900; Thermo Fisher Scientific) plus halt protease inhibitor cocktail (87787; Thermo Fisher Scientific) for 30 min on ice. After addition of 4× Laemmli sample buffer (Bio‐Rad), samples were separated by 10% SDS‐PAGE electrophoresis, transferred to nitrocellulose membrane (Bio‐Rad), and proteins of interest were incubated overnight with diluted primary antibodies (CD206, 1:1000, 24595, Cell signaling Technology; NLRP3, 1:1000, 15101, Cell signaling Technology; iNOS, 1:1000, PA5‐17106, Invitrogen; Ym1, 1:1000, AF2446, R&D system; ICAM‐1, 1:1000, ab222736, Abcam; IL‐1β, 1:1000, 12703, Cell signaling Technology; NF‐κB p65, 1:1000, 8242, Cell signaling Technology; Lamin A/C, 1:1000, 2032, Cell signaling Technology and β‐actin, 1:2500, 4970, Cell signaling Technology) and then incubated for 1 h with a secondary antibody (Invitrogen). The membranes were visualized using enhanced chemiluminescence (1705061; Bio‐Rad) within the luminescent image analyzer (Thermo Fisher Scientific).

### Immunohistochemical Analysis

Formalin‐fixed, paraffin‐embedded lung tissue sections were stained with the Anti‐Ym1 antibody (ab230610; Abcam), and paraffin‐embedded heart, liver, liver, spleen, lung, and kidney tissue sections were stained with the Anti‐ICAM‐1 antibody (ab222736; Abcam) by the In‐Vivo Animal Core at the University of Michigan. Digital images of the stained tissue sections were acquired using a KEYENCE BZ‐X800 microscope.

### Immunocytochemistry Analysis

For NF‐κB p65 nuclear/cytosolic localization analysis, BMDMs were seeded on culture slides (354105, Corning) and incubated overnight. Subsequently, BMDMs were blocked with a solution containing 5% BSA (Thermo Fisher Scientific) and 0.05% Triton X‐100 (Thermo Fisher Scientific) in PBS for 1 h. Immunostaining was conducted using an anti‐NF‐κB p65 antibody (1:100, 8242; Cell signaling Technology) overnight at 4 °C, followed by incubation with a second antibody (ab150064; Abcam). Nuclear staining was achieved using DAPI (62248; Thermo Fisher Scientific). Microscopic imaging was performed using a Nikon A1si confocal microscope. For ICAM‐1 expression analysis, RAW 264.7 cells were subjected to blocking and then incubated with PE anti‐mouse CD54 Antibody (1:100, 116107; Biolegend) overnight at 4 °C. The sections were rinsed twice to remove any unbound antibodies before imaging with a KEYENCE BZ‐X800 microscope.

### Immunoprecipitation

Immunoprecipitation was conducted in accordance with the manufacturer's instructions provided in the classic magnetic IP/Co‐IP kit manual (88804, Thermo Fisher Scientific). Cells were washed once with ice‐cold PBS and were lysed in IP lysis buffer with protease inhibitor cocktail, incubated on ice for 10 min, and centrifuged at 13000 rcf at 4 °C for 10 min. The supernatant was incubated with indicated antibodies (2 µg per sample) overnight at 4 °C and then incubated with Protein A/G Agarose for 1 h at room temperature. The bound proteins were eluted by incubating with elution buffer for 10 min and then boiling in 4× loading buffer and subjected to Western blotting.

### Nuclear‐Cytosolic Extraction

Nuclear and cytosolic extracts were prepared as per the manufacturer's protocol using the NE‐PER kit (78833, ThermoFisher Scientific). Briefly, cells were lysed in Cytoplasmic Extraction Reagent I (CER I, 200 µL) buffer supplemented with a protease inhibitor cocktail to extract cytosolic proteins. Following the addition of Cytoplasmic Extraction Reagent II (CER II, 11 µL) buffer, the cytosolic fraction was obtained by centrifugation at 16 000 rcf for 10 min at 4 °C. The resulting pellet containing nuclei was then resuspended in Nuclear Extraction Reagent (NER, 100 µL) buffer supplemented with a protease inhibitor cocktail. Subsequent centrifugation at 16 000 rcf for 10 min at 4 °C yielded the nuclear protein extract in the supernatant.

### Identification of Citrullination Sites by LC‐MS/MS

BALF samples from three mice per group were pooled to obtain samples (*n* = 3 mice per sample) for BALF cell isolation. The samples were centrifuged at 400 rcf at 4 °C for 5 min to pellet the cells, which were then treated with RBC lysis buffer (eBioscience) to remove red blood cells. The isolated BALF cells were suspended in RIPA buffer containing a protease inhibitor cocktail and heated at 95 °C for 15 min. Protein concentration was determined using Qubit fluorometry (Invitrogen). Subsequently, 10 µg of each sample was subjected to SDS‐PAGE using 10% Bis‐Tris NuPage Mini‐gel (Invitrogen) with the MES buffer system. Gel electrophoresis was performed for 2 cm, and the gel was then cut into ten equally sized bands. In‐gel digestion of each band was carried out with trypsin using a robot (DigestPro, CEM), involving washing with 25 mm ammonium bicarbonate followed by acetonitrile, reduction with 10 mm dithiothreitol at 60 °C, alkylation with 50 mm iodoacetamide at room temperature, and digestion with sequencing grade trypsin (Promega) at 37 °C for 4 h. The digestion process was quenched with formic acid, and the supernatant was directly analyzed. Half of each digested sample was subjected to nano LC‐MS/MS using a Waters M‐Class LC system coupled to a ThermoFisher Exploris 480 mass spectrometer. Peptides were loaded onto a trapping column and eluted over a 75 µm analytical column packed with XSelect CSH C18 resin (Waters). The column temperature was maintained at 55 °C using a column heater (Sonication), and the mass spectrometer operated in data‐dependent mode with the Orbitrap set at 60000 FWHM and 15000 FWHM for MS and MS/MS, respectively, with a 3‐s cycle for both MS and MS/MS. Each sample was analyzed for 5 h. Mascot with Trypsin/P enzyme parameters was used for data search against the SwissProt Mouse database appended with *Pseudomonas aeruginosa* BL04. A differential modification of 0.984 on arginine was specified to account for citrullination by PAD2. Data were filtered and analyzed using Scaffold 5 proteome software (version: 5.3.3).

### Establishment of Mouse R171A RAW 264.7 Stable Cell Lines

Subcloning, lentivirus production, transduction, and cell selection were performed by the University of Michigan Vector Core. A geneblock encoding mouse Rela, with arginine 171 mutated to alanine (R171A) and an HA tag fused to the C‐terminus through a 4‐amino acid linker, was synthesized by Twist Biosciences. This Rela R171A‐HA geneblock was then cloned into the NheI/XhoI‐digested pLentiLox CAG‐mcs‐mPGK‐meGFP T2a Puromycin lentiviral vector. DNA preparation for lentiviral production was carried out using the Qiagen Plasmid Plus Midiprep DNA kit (Qiagen) following the manufacturer's instructions. Lentivirus packaging vectors psPAX2 and pC1‐VSVG were co‐transfected with either pLentilox CAG‐Rela R171A‐HA (for R171A mutant) or mPGK‐GFP/puro (for WT) plasmid using standard PEI precipitation methods. PEI precipitation involved incubating the plasmids with PEI (molecular weight 2500, Polysciences, Inc) in OptiMEM (Life Technologies) at room temperature for 20 min before adding to DMEM with 10% FBS media. This DNA/PEI‐containing media was added to transfect 293T cells (ATCC). Culture supernatant was collected after 72 h, supplemented with 8 µg mL^−1^ polybrene (Sigma), and applied to RAW 264.7 cells. Cells were then incubated at 37 °C, 5% CO_2_ for 48 h before undergoing puromycin selection for 5 days.

### Quantification of AFM41a Using High‐Performance Liquid Chromatography (HPLC)

HPLC was performed using a Thermofisher Vanquish Core LC system equipped with a 3 µm C18 reversed‐phase column (4.6 × 150 mm) and a UV–Vis detector set at λ = 220 nm for AFM41a detection. AFM41a was dissolved in water (1 mg mL^−1^), and HPLC samples were prepared by mixing 800 µL of the aqueous sample with 200 µL of acetonitrile. The mobile phase was an isocratic mixture of 20% acetonitrile and 80% water (ACN–H₂O) with 0.1% trifluoracetic acid, ensuring optimal separation of AFM41a. The flow rate was maintained at 0.5 mL min^−1^, and the column temperature was held at 30 °C. Chromatograms were analyzed to assess peak area, and a standard curve was generated by titrating different AFM41a concentrations to establish a linear correlation between peak area and drug concentration. This standard curve was then used to quantify drug loading efficiency by measuring AFM41a concentrations in the supernatant before and after conjugation to AuNPs. The difference between pre‐ and post‐conjugation supernatant concentrations was used to calculate the amount of AFM41a successfully loaded onto the AuNP surface.

### Statistical Analysis

The statistical analysis was performed using GraphPad Prism 8 software. For comparisons involving three or more groups, the one‐way or two‐way ANOVA with Turkey's multiple comparisons test was applied to compare individual groups. For comparisons between the two groups, an unpaired *t*‐test was utilized. A significance level of *p*‐value < 0.05 was set to determine statistical significance, indicating meaningful differences between the groups.

## Conflict of Interest

The authors declare no conflict of interest.

## Author Contributions

X.Y. and Y.S. contributed equally to this work. X.Y., Y.S., K.K., and Y.L. conceptualized the study. X.Y., Y.S., T.D., O.W., L.S., L.B., P.R., and Y.L. developed the methodology. X.Y., Y.S., and Y.L. performed the formal analysis. X.Y., Y.S., T.D., O.W., L.S., J.M., M.Z., and C.Q carried out the investigation. X.Y. and Y.S. wrote the original draft. X.Y., Y.S., Y.L. K.K., and J.M. wrote, reviewed, and edited the final draft. X.Y., Y.S., K.K., and Y.L. visualized the study. K.K. and Y.L. supervised the study. Y.L. and K.K. administered the project. Y.L. acquired funds.

## Supporting information



Supporting Information

## Data Availability

The data that support the findings of this study are available from the corresponding author upon reasonable request.
