# Supplementary material for: Citrullination of NF‐κB p65 by PAD2 as a Novel Therapeutic Target for Modulating Macrophage Polarization in Acute Lung Injury
Source: Adv Sci (Weinh). 2025 Mar 14;12(18):2413253. doi: 10.1002/advs.202413253 (PMC12079445; doi:10.1002/advs.202413253)
Supplement: Supplementary file 1 — Supporting Information [file ADVS-12-2413253-s001.pdf]

ADVANCED SCIENCE
Open Access

Supporting Information

for *Adv. Sci.*, DOI 10.1002/advs.202413253

Citrullination of NF-κB p65 by PAD2 as a Novel Therapeutic Target for Modulating Macrophage Polarization in Acute Lung Injury

Xin Yu, Yujing Song, Tao Dong, Wenlu Ouyang, Chao Quan, Liujiazi Shao, Leonard Barasa, Paul R. Thompson, Mao Zhang, Jianjie Ma, Katsuo Kurabayashi and Yongqing Li**

Supporting information

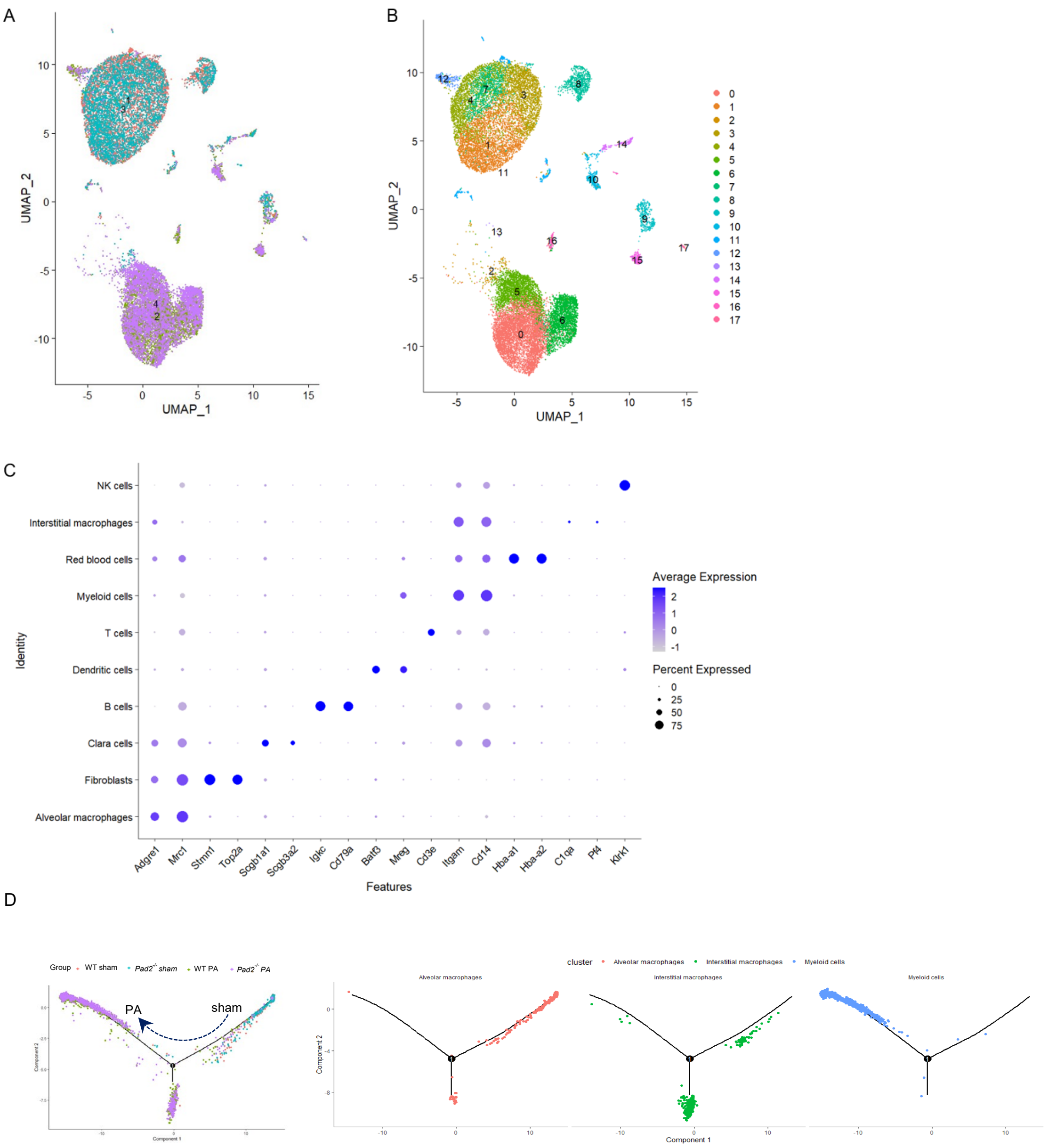

Figure S1: scRNA-seq analysis of cell clusters from WT and *Pad2*$^{-/-}$ mice in alveoli

A. UMAP plots showing all BALF cells from WT and *Pad2*$^{-/-}$ mice. The plots represent the following groups: 1, WT sham; 2, WT PA; 3, *Pad2*$^{-/-}$ sham; and 4, *Pad2*$^{-/-}$ PA. BALF cells from three mice were pooled to create one sample for each group.

B. UMAP plots displaying 22,193 cells categorized into 18 clusters from WT and *Pad2*$^{-/-}$ mice, comparing PA-induced sepsis and sham groups.

C. Expression levels of marker genes across ten distinct clusters of BALF cells from WT and *Pad2*$^{-/-}$ mice, illustrated by dot plots. The brightness of the circles represents log-normalized average expression, while the circle size indicates the percentage of cells expressing the gene.

D. The Monocle prediction of the sham-PA developmental trajectory from WT sham, WT PA, *Pad2*$^{-/-}$ sham and *Pad2*$^{-/-}$ PA groups (Left panel). And the monocle prediction of myeloid cell-macrophage developmental trajectory with each cell cluster shown separately (Right panel).

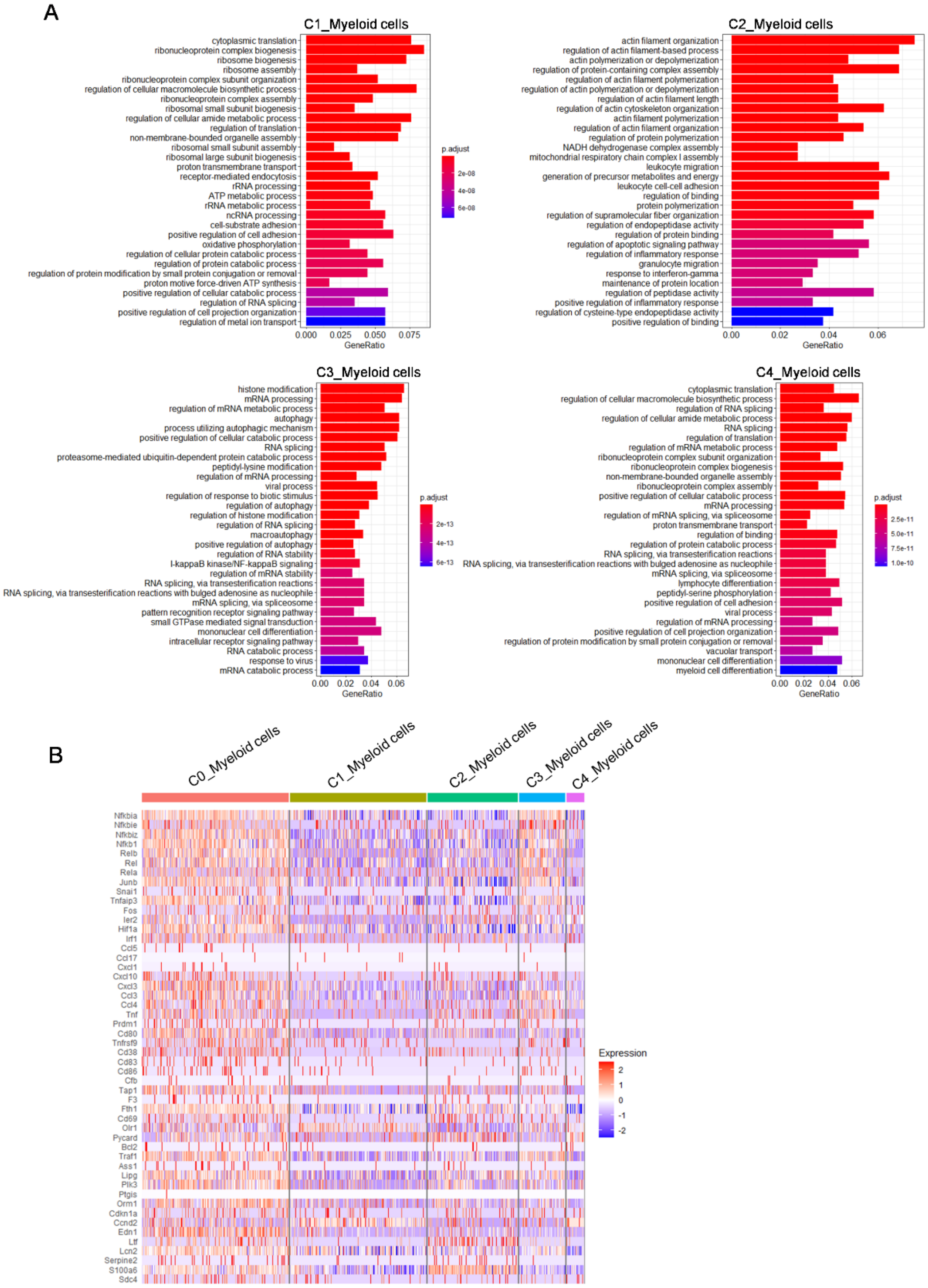

Figure S2: Analysis of Myeloid Cell Clusters and NF-κB Signaling

A. Enrichment analysis of representative GO biological pathways for Cluster 1, 2, 3, and 4 myeloid cells, illustrated by bar plots.

B. Heatmap showing NF-κB signaling gene expression within Cluster 0, 1, 2, 3, and 4 myeloid cells, annotated by cluster types.

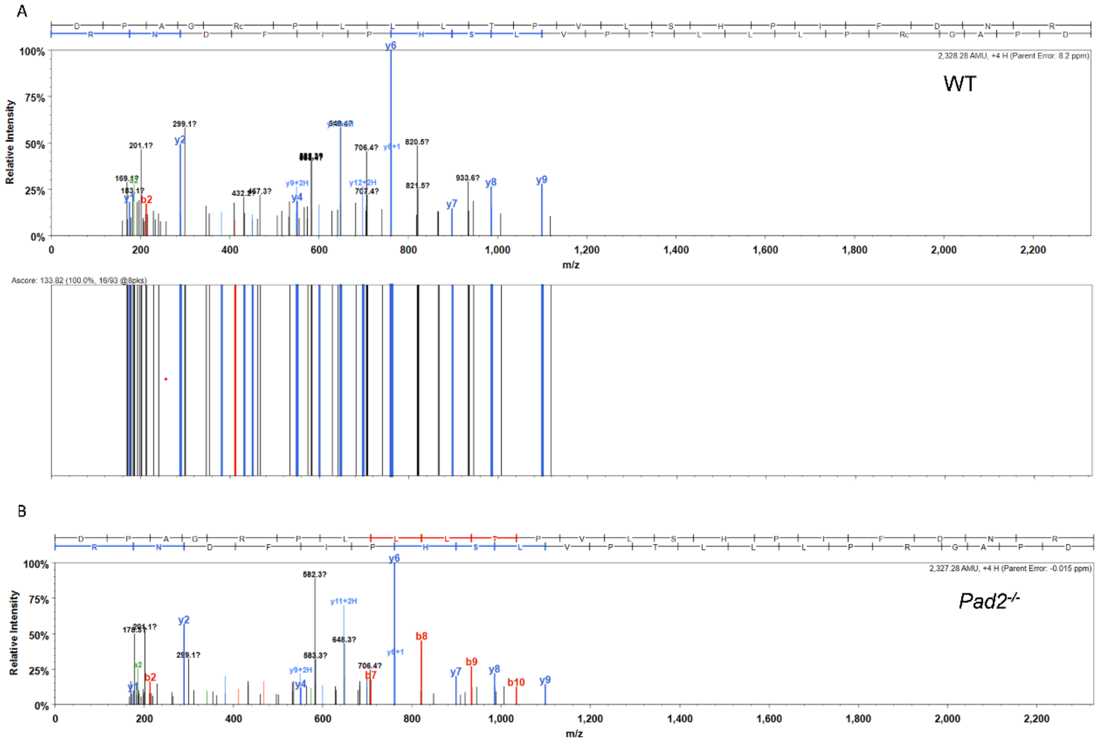

C

WT

B	B Ions	B+2H	B-NH3	B-H2O	AA	Y Ions	Y+2H	Y-NH3	Y-H2O	Y
1	116.0	58.5		98.0	D	2,329.3	1,165.1	2,312.2	2,311.3	21
2	213.1	107.0		195.1	P	2,214.2	1,107.6	2,197.2	2,196.2	20
3	284.1	142.6		266.1	A	2,117.2	1,059.1	2,100.2	2,099.2	19
4	341.1	171.1		323.1	G	2,046.1	1,023.6	2,029.1	2,028.1	18
5	498.2	249.6	481.2	480.2	R+1	1,989.1	995.1	1,972.1	1,971.1	17
6	595.3	298.1	578.3	577.3	P	1,832.0	916.5	1,815.0	1,814.0	16
7	708.4	354.7	691.3	690.4	L	1,735.0	868.0	1,718.0	1,717.0	15
8	821.5	411.2	804.4	803.4	L	1,621.9	811.5	1,604.9	1,603.9	14
9	934.5	467.8	917.5	916.5	L	1,508.8	754.9	1,491.8	1,490.8	13
10	1,035.6	518.3	1,018.6	1,017.6	T	1,395.7	698.4	1,378.7	1,377.7	12
11	1,132.6	566.8	1,115.6	1,114.6	P	1,294.7	647.8	1,277.7	1,276.7	11
12	1,231.7	616.4	1,214.7	1,213.7	V	1,197.6	599.3	1,180.6	1,179.6	10
13	1,344.8	672.9	1,327.8	1,326.8	L	1,098.6	549.8	1,081.5	1,080.6	9
14	1,431.8	716.4	1,414.8	1,413.8	S	985.5	493.2	968.5	967.5	8
15	1,568.9	784.9	1,551.9	1,550.9	H	898.5	449.7	881.4	880.4	7
16	1,665.9	833.5	1,648.9	1,647.9	P	761.4	381.2	744.4	743.4	6
17	1,779.0	890.0	1,762.0	1,761.0	I	664.3	332.7	647.3	646.3	5
18	1,926.1	963.5	1,909.1	1,908.1	F	551.3	276.1	534.2	533.2	4
19	2,041.1	1,021.1	2,024.1	2,023.1	D	404.2	202.6	387.2	386.2	3
20	2,155.2	1,078.1	2,138.1	2,137.1	N	289.2	145.1	272.1		2
21	2,329.3	1,165.1	2,312.2	2,311.3	R	175.1	88.1	158.1		1

Pad2-/-

B	B Ions	B+2H	B-NH3	B-H2O	AA	Y Ions	Y+2H	Y-NH3	Y-H2O	Y
1	116.0	58.5		98.0	D	2,328.3	1,164.6	2,311.3	2,310.3	21
2	213.1	107.0		195.1	P	2,213.3	1,107.1	2,196.2	2,195.2	20
3	284.1	142.6		266.1	A	2,116.2	1,058.6	2,099.2	2,098.2	19
4	341.1	171.1		323.1	G	2,045.2	1,023.1	2,028.1	2,027.2	18
5	497.2	249.1	480.2	479.2	R	1,988.1	994.6	1,971.1	1,970.1	17
6	594.3	297.7	577.3	576.3	P	1,832.0	916.5	1,815.0	1,814.0	16
7	707.4	354.2	690.4	689.4	L	1,735.0	868.0	1,718.0	1,717.0	15
8	820.5	410.7	803.4	802.5	L	1,621.9	811.5	1,604.9	1,603.9	14
9	933.6	467.3	916.5	915.5	L	1,508.8	754.9	1,491.8	1,490.8	13
10	1,034.6	517.8	1,017.6	1,016.6	T	1,395.7	698.4	1,378.7	1,377.7	12
11	1,131.7	566.3	1,114.6	1,113.6	P	1,294.7	647.8	1,277.7	1,276.7	11
12	1,230.7	615.9	1,213.7	1,212.7	V	1,197.6	599.3	1,180.6	1,179.6	10
13	1,343.8	672.4	1,326.8	1,325.8	L	1,098.6	549.8	1,081.5	1,080.6	9
14	1,430.8	715.9	1,413.8	1,412.8	S	985.5	493.2	968.5	967.5	8
15	1,567.9	784.5	1,550.9	1,549.9	H	898.5	449.7	881.4	880.4	7
16	1,664.9	833.0	1,647.9	1,646.9	P	761.4	381.2	744.4	743.4	6
17	1,778.0	889.5	1,761.0	1,760.0	I	664.3	332.7	647.3	646.3	5
18	1,925.1	963.1	1,908.1	1,907.1	F	551.3	276.1	534.2	533.2	4
19	2,040.1	1,020.6	2,023.1	2,022.1	D	404.2	202.6	387.2	386.2	3
20	2,154.2	1,077.6	2,137.1	2,136.2	N	289.2	145.1	272.1		2
21	2,328.3	1,164.6	2,311.3	2,310.3	R	175.1	88.1	158.1		1

Figure S3: Annotated MS fragmentation spectra of the citrullinated R171 peptides of NF-kB p65 in BALF cells from WT and *Pad2*$^{-/-}$ mice. The MS spectra of the WT group (**S3A, upper panel**) and *Pad2*$^{-/-}$ group (**S3B**) were exported from Scaffold PTM software, along with the related fragmentation tables of the WT group (**S3C, left panel**) and *Pad2*$^{-/-}$ group **(S3C, right panel)**. The Ambiguity Score (A-score) measures the probability of correct modification of citrullination site location within the peptide based on the presence and intensity of site-determining peaks in the MS/MS spectrum, with an A-score of >13 considered significant. The R171 peptide of the WT group shows an A-score of 133.82, confirming the existence of the citrullinated R171 site in the presence of PAD2 enzyme (**S3A, lower panel**). However, the R171 peptide was not modified by citrullination after PAD2 deficiency and lacked an A-score.

A

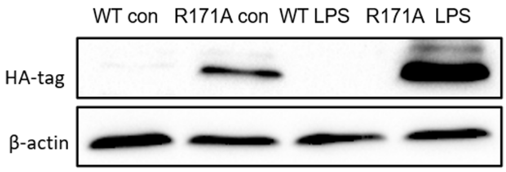

B

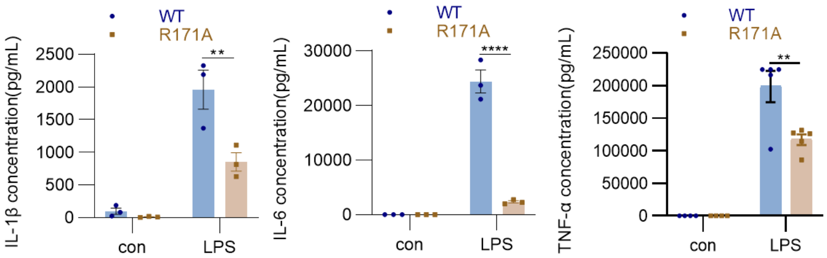

Figure S4: Validation of R171A mutant RAW264.7 cells.

A. Western blot analysis of HA-tag protein in R171A mutant RAW 264.7 cells and WT cells, stimulated with PA-derived LPS (250 ng/mL, 24 hours) and control.

B. ELISA results showing levels of IL-1β, IL-6 and TNF-α in cell culture supernatant from R171A mutant RAW 264.7 cells and WT cells stimulated with PA-derived LPS (250 ng/mL, 24 hour) and control (n = 3-5). Data for all bar charts were analyzed using two-way ANOVA. Data are presented as means ± SEM. Asterisks (*) denote statistical significance, with P-values indicated as follows: *, P<0.05; **, P < 0.01.

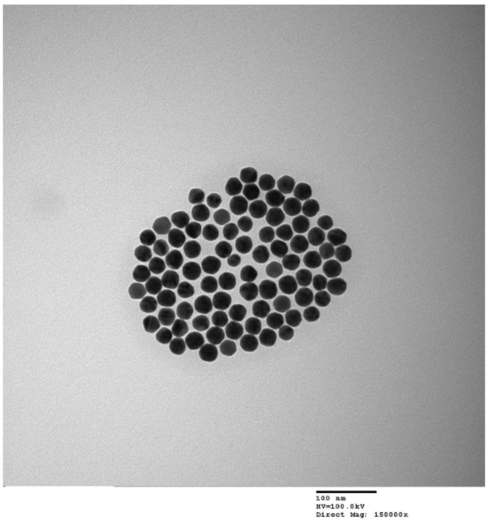

Figure S5: Characterization of gold nanoparticles. Transmission electron microscopy (TEM) image of gold nanoparticles (AuNPs).

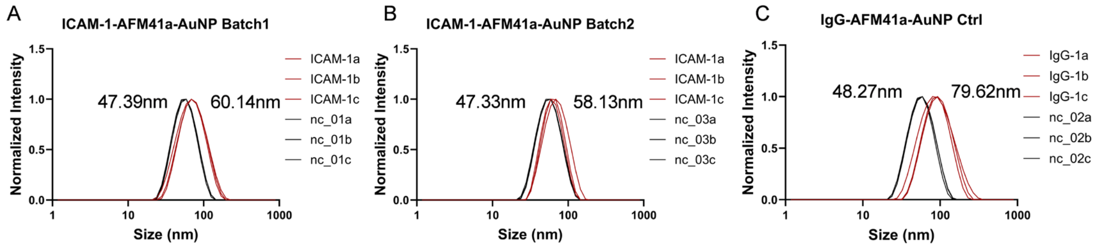

D

AuNP	State	Size (nm)	Polydispersity Index (PDI)	Zeta Potential (mV)
ICAM-1-AFM41a-AuNP (Batch1)	Before	47.39	0.2051	-17.07
	After	60.14	0.199	19.22
ICAM-1-AFM41a-AuNP (Batch 2)	Before	47.33	0.2002	-11.2
	After	58.13	0.2234	17.4
IgG-AFM41a-AuNP (Ctrl)	Before	48.27	0.2057	-23.57
	After	79.62	0.226	18.58

Figure S6: Characterization of Nanoparticle Size and Surface Charge.

A, B. Dynamic light scattering (DLS) measurements of ICAM-1-AFM41a-AuNPs from Batch 1 (A) and Batch 2 (B), conducted before and after conjugation with anti-ICAM-1 antibody and AFM41a.

C. DLS measurements of the IgG-AFM41a-AuNP control, performed before and after conjugation with anti-IgG antibody and AFM41a.

D. A summary of size (nm), polydispersity index (PDI), and zeta potential (mV) for ICAM-1-AFM41a-AuNPs (Batch 1), ICAM-1-AFM41a-AuNPs (Batch 2), and the IgG-AFM41a-AuNP control, both before and after conjugation with the relevant antibodies and AFM41a.

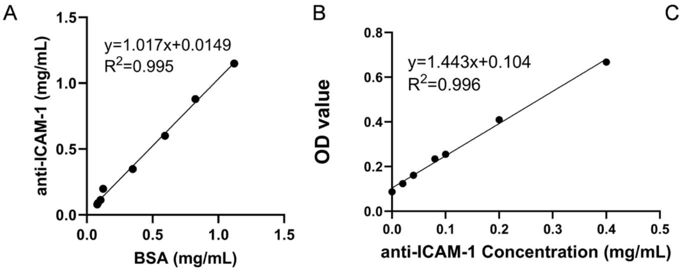

C

Anti-ICAM-1	Concentration (mg/mL)	Average (mg/mL)	Loading (Abs/NPs)
Before	0.107	0.108	**93.1**
	0.107		
	0.110		
After	0.091	0.090	
	0.091		
	0.087		

Figure S7: Characterization of Anti-ICAM-1 and IgG Control Loading Efficiency Using Bicinchoninic Acid (BCA) Assay.

A. Correlation between anti-ICAM-1 and the BCA assay standard, bovine serum albumin (BSA). The high correlation coefficient (R^2 = 0.995, k = 1.017) indicates that the anti-ICAM-1 concentration accurately aligns with the BSA standard, validating the suitability of the BCA assay for quantifying the anti-ICAM-1 concentration.

B. Standard curve of the anti-ICAM-1 BCA assay, demonstrating a linear dynamic range from 0.4 mg/mL to 0.02 mg/mL. This ensures precise quantification of anti-ICAM-1 concentration before and after conjugation.

C. Quantification of anti-ICAM-1 loading efficiency on the AuNP surface using the BCA assay. The anti-ICAM-1 loading efficiency was calculated by subtracting the post-conjugation supernatant concentration from the pre-conjugation supernatant concentration, both of which were quantified using the BCA assay standard curve above. The starting AuNP concentration was 0.15 mg/mL (2.33 $\times 10^{11}$ nps/mL). The initial anti-ICAM-1 concentration before conjugation was 0.108 mg/mL, while the remaining concentration in the supernatant after conjugation was 0.09 mg/mL. Based on this difference, the conjugated antibody molecules per AuNP were calculated to be 93.1 Ab/NPs, confirming effective surface functionalization. All measurements were performed in triplicate to ensure reproducibility.

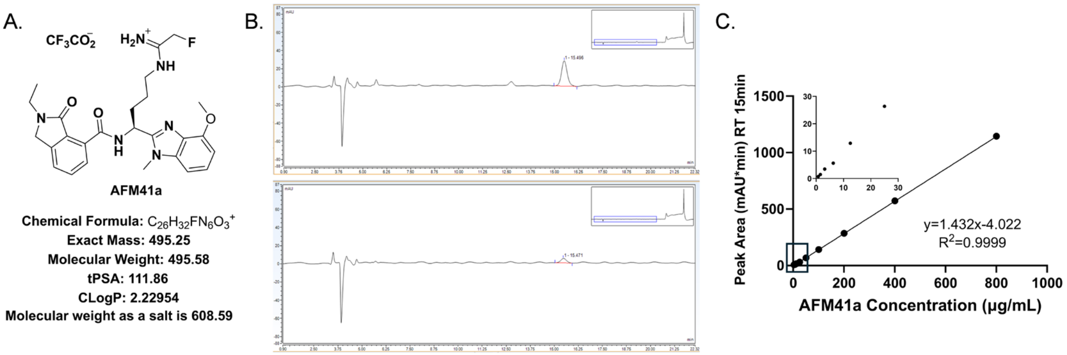

D.

AFM41a	Retention Time (min)	Peak Area (mAU*min)	Concentration (µg/mL)	Average (µg/mL)	Loading (drug/NPs)
Batch1 Before	15.546	10.6007	12.84	12.84	**6572.3**
Batch1 After	15.496	9.7149	11.79	11.58	
	16.154	9.3517	11.36		
Batch 2 Before	15.554	3.0183	3.89	3.89	**7437.5**
Batch 2 After	15.471	1.7101	2.35	2.47	
	16.246	1.9107	2.59		

Figure S8: Characterization of AFM41a Loading Efficiency Using High-Performance Liquid Chromatography (HPLC).

A. Chemical structure and molecular properties of AFM41a, with an exact mass of 495.25 Da and a molecular weight of 608.59 Da in its salt form.

B. HPLC chromatogram of AFM41a detected in the supernatant for two batches of AuNP conjugation, with a prominent peak at 15 min, indicating the presence of unbound AFM41a (as marked in red). Chromatography was performed using a Thermo Fisher Vanquish Core LC system, equipped with a 3 µm C18 reverse-phase column (4.6 × 150 mm) and a UV-Vis detector set at λ = 220 nm for AFM41a detection. AFM41a was dissolved in water, and HPLC samples were prepared by mixing 800 µL of the aqueous sample with 200 µL of acetonitrile. The injection volume was 20 µL per run. An isocratic mobile phase consisting of 80% acetonitrile and 20% water (ACN–H_2O) with 0.1% trifluoroacetic acid (TFA) was used at a flow rate of 0.5 mL/min.

C. AFM41a standard curve, generated by titrating different concentrations and assessing peak area to establish a linear dynamic range from 800 µg/mL to 0.781 µg/mL. The standard curve was used to quantify the supernatant AFM41a concentration, and the drug loading efficiency of AFM41a was calculated by subtracting the post-conjugation supernatant concentration from the pre-conjugation supernatant concentration.

D. Quantification of AFM41a loading efficiency on the AuNPs. The starting AuNP concentration was 0.15 mg/mL (2.33×10^{11} nps/mL). The initial AFM41a concentrations before conjugation were 12.84 µg/mL and 3.89 µg/mL, while the remaining concentrations in the supernatant after conjugation were 11.58 µg/mL and 2.47 µg/mL, respectively. Based on this difference, the conjugated AFM41a molecules per AuNP were calculated to be 6572.3 Drug/NPs and 7437.2 Drug/NPs, confirming successful drug loading. The measurements were performed in duplicate using the HPLC assay to ensure reproducibility.

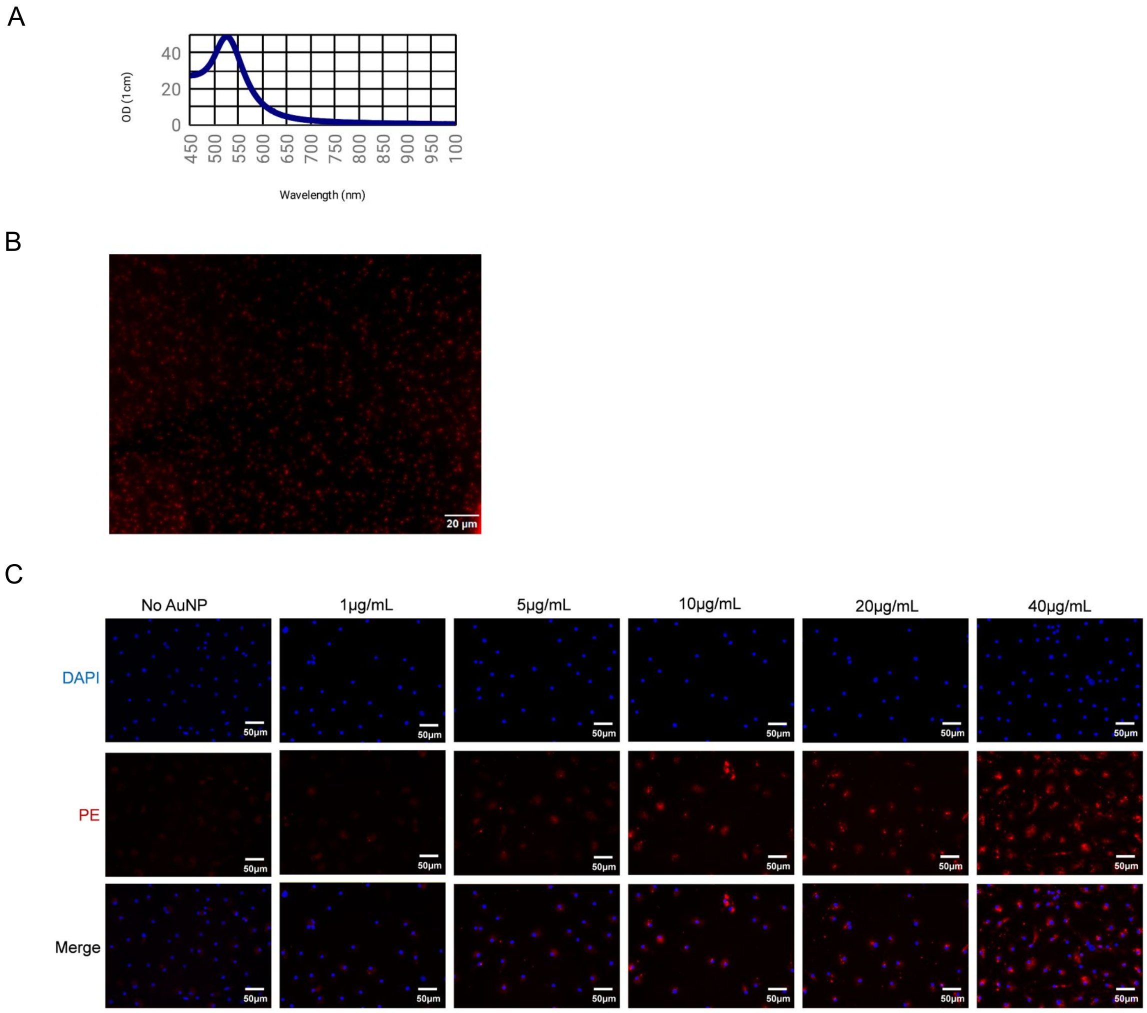

Figure S9: AuNP uptake in LPS-stimulated macrophages.

A. Absorbance spectrum of the AuNP colloidal solution.

B. Microscopic image of AuNPs labeled with PE-conjugated anti-rat IgG. Scale bars represent 20 µm.

C. BMDMs were pre-treated with PA-derived LPS (250 ng/mL, 6 hours), then treated with or without PE-anti-ICAM-1-AFM41a-loaded AuNPs (1 µg/mL, 5 µg/mL, 10 µg/mL, 20 µg/mL, 40 µg/mL) for 3 hours. Cells were analyzed by immunofluorescence to assess the uptake of AuNPs. Scale bars represent 50 µm.

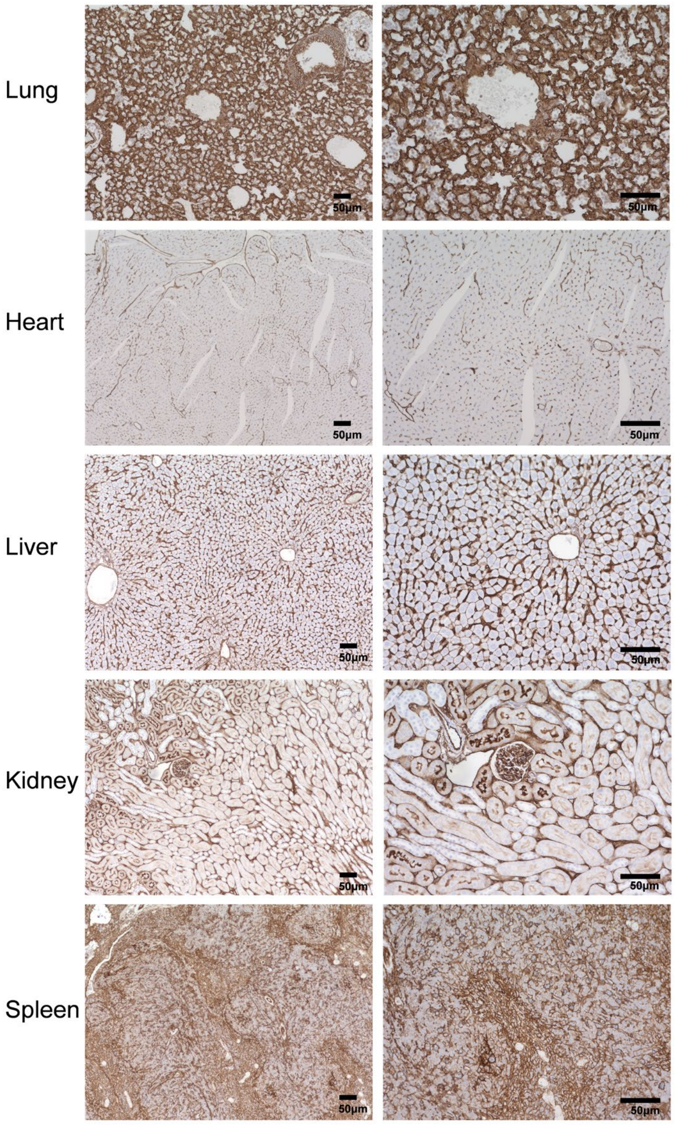

Figure S10: ICAM-1 expression in various organs from PA-infected mice. IHC staining for ICAM-1 in lung, heart, liver, kidney, and spleen tissues of mice with PA-induced ALI. Scale bars represent 50 μm.
